# Application of a Web-based Self-assessment Triage Tool During the COVID-19 Pandemic: Descriptive Study

**DOI:** 10.2196/34134

**Published:** 2022-04-04

**Authors:** Anna Nowicka, Jakub Jaszczak, Anna Szymanek Pasternak, Krzysztof Simon

**Affiliations:** 1 Provincial Specialist Hospital them. J. Gromkowski Wrocław Poland; 2 Infermedica Wrocław Poland; 3 Department of Infectious Diseases and Hepatology Wroclaw Medical University Wrocław Poland

**Keywords:** COVID-19, symptom checker, preclinical triage, self-assessment tool, online applications, COVID-19 remote triage, COVID-19 self-assessment

## Abstract

**Background:**

The COVID-19 pandemic has sped up the implementation of telehealth solutions in medicine. A few symptom checkers dedicated for COVID-19 have been described, but it remains unclear whether and how they can affect patients and health systems.

**Objective:**

This paper demonstrates our experiences with the COVID-19 risk assessment (CRA) tool. We tried to determine who the user of the web-based COVID-19 triage app is and compare this group with patients in the infectious diseases ward’s admission room to evaluate who could benefit from implementing the COVID-19 online symptom checker as a remote triage solution.

**Methods:**

We analyzed the answers of 248,862 people interacting with an online World Health Organization–based triage tool for assessing the probability of SARS-CoV-2 infection. These users filled in an online questionnaire between April 7 and August 6, 2020. Based on the presented symptoms, risk factors, and demographics, the tool assessed whether the user’s answers were suggestive of COVID-19 and recommended appropriate action. Subsequently, we compared the sociodemographic and clinical characteristics of tool users with patients admitted to the Infectious Diseases Admission Room of J. Gromkowski Hospital in Wrocław.

**Results:**

The CRA tool tended to be used by asymptomatic or oligosymptomatic individuals (171,226 [68.80%] of all users). Most users were young (162,432 [65.27%] were below 40 years of age) and without comorbidities. Only 77,645 (31.20%) of the self-assessment app users were suspected of COVID-19 based on their reported symptoms. On the contrary, most admission room patients were symptomatic—symptoms such as fever, cough, and dyspnea were prevalent in both COVID-19-positive and COVID-19-negative patients. COVID-19-suspected patients in the CRA tool group presented similar COVID-19 symptoms as those who presented to the admission room. These were cough (25,062/40,007 [62.64%] in the CRA tool group vs 138/232 [59.48%] in the admission room group), fever (23,123/40,007 [57.80%] in the CRA tool group vs 146/232 [62.93%] in the admission room group), and shortness of breath (15,157/40,007 [37.89%] in the CRA tool group vs 87/232 [37.50%] in the admission room group).

**Conclusions:**

The comparison between the symptomatology of the users interacting with the CRA tool and those visiting the admission room revealed 2 major patient groups who could have benefited from the implementation of the self-assessment app in preclinical triage settings. The primary users of the CRA tool were young, oligosymptomatic individuals looking for screening for COVID-19 and reassurance early in the COVID-19 pandemic. The other group were users presenting the typical symptoms suggestive of COVID-19 at that time. The CRA tool recognized these individuals as potentially COVID-19 positive and directed them to the proper level of care. These use cases fulfil the idea of preclinical triage; however, the accuracy and influence on health care must be examined in the clinical setting.

## Introduction

### Background

After the outbreak of the COVID-19 pandemic, the health care systems of affected countries faced an unprecedented challenge. Ensuring the continuity of care and screening the vast number of suspected patients have put a significant strain on health care, leading to the depletion of public health resources [1,2]. Although the health system resources were transferred to provide critical services to patients suffering from COVID-19, the utilization of medical visits reduced by even 42% [2], suggesting that patients with less severe illnesses tended to avoid in-person consultation or had no possibility to attend one.

During the pandemic, especially in the early days, there was much uncertainty regarding the symptomatology and clinical course of the novel coronavirus disease. This has been reflected in the number of searches for the phrase “covid 19 symptoms” on the Google platform, which at the time of the study varied from 443,000 to 2.2 million searches per month just for the United States [3].

These uncertain times have presented an opportunity to popularize telehealth solutions in medicine. The means of remote consultations have found their way mostly in primary care as a substitute for in-person visits [4] but also as a way of remote triage of COVID-19 patients.

Triage is defined as a classification of patients according to their urgencies. Remote triage uses the means of distance communication, such as telephones or interactive websites, allowing for the segregation of patients before they interact with health care professionals. Remote triage solutions have been proven helpful in telephone call centers, where they have been associated with lower in-person health care use [5]. They have also been demonstrated to be useful in the triage of COVID-19 patients, as they have reduced the number of unnecessary consultations, hence reducing the exposure of the staff to COVID-19 [6]. Web-based COVID-19 symptom checkers and triage tools have also proved useful in scheduling tests [7,8], monitoring symptoms [9-11], providing evidence-based educational value [8,9,12], and supporting self-isolation [13].

### Objective

In this study, we wanted to share our findings regarding the COVID-19 risk assessment (CRA) tool. It was a World Health Organization (WHO) guidelines-based online triage tool, which assessed the risk of SARS-CoV-2 infection and returned a probable outcome with a concise recommendation of what to do next, along with evidence-based educational materials about COVID-19.

We gathered and analyzed the data of 651,757 patients interacting with the CRA tool, focusing on their demographics, risk factors, reported symptoms, possible exposure to SARS-CoV-2, and recommended triage. The aim was to establish who the main users of web-based COVID-19 symptom checkers (age, sex, comorbidities, presenting symptoms) are and who might have benefitted from implementing COVID-19 symptom checkers as preclinical triage solutions.

Since confirming the diagnosis in an online self-assessment tool was not achievable, we compared the results (sociodemographic and clinical characteristics of CRA users) with the health records of the Infectious Diseases Admission Room of J. Gromkowski Hospital in Wrocław to establish whether and how these groups corresponded. The goal was to evaluate who could benefit from implementing this solution as preclinical triage.

## Methods

### Study Population

Since April 7, 2020, we have been collecting and utilizing responses from the CRA tool users. The app was developed by Infermedica company, as a non-profit project. It utilized a diagnostic algorithm designed based on WHO and Centers for Disease Control and Prevention (CDC) recommendations. The specific time frame was chosen due to periodical updates of the app questions flow. In the selected period, there were no major changes to the question flow so that the collected information could be unbiased.

### Inclusion Criteria

The study population included individuals concerned about their risk of COVID-19 infection:

- Users who filled the questionnaire available through the Infermedica website between April 7 and August 6, 2020
- Users who filled the questionnaires available on third-party websites, which obtained permission to use our tool within their platforms between April 7 and August 6, 2020

### Exclusion Criteria

The exclusion criteria were:

- Completing the interview in an outdated 1.0 and 2.0 version (not all providers of our tool updated their software before the beginning of the study)
- Completing the interview in a version customized for a national health system so that it was incompatible with WHO and CDC recommendations
- Not completing the whole interview
- Age below 18 years
- Completing the interview in a language other than Polish

### Data Privacy and Ethical Statement

The study population consisted of 2 arms: users of the web app and patients in the admission room.

The app arm consisted of users of the web app who accepted the terms of service. All data processed through the COVID-19 risk assessment checker were anonymous and did not allow us to identify an individual based on the information provided during the interview. Informed consent to use anonymized data was provided by the users by accepting the terms of service. A privacy policy and personal data protection were applied.

The admission room arm of the study did not require ethics committee approval as a retrospective study, according to the guidelines of the local ethical compliance body [14].

### COVID-19 Symptom Checker Characteristics

The CRA is a triage tool dedicated to nonprofessional users. The checkup was designed to assess whether the user’s symptoms may be the result of SARS-CoV-2 infection. It had a form of a responsive web app that could be embedded within a website or an Application Program Interface (API) that can serve as a technological core for building custom apps. (An API is a set of routines, protocols, and tools for building software applications. Basically, an API specifies how software components should interact. It serves as a technological core for custom-building applications.) The flow of the interview was solely based on the official WHO guidelines for diagnosing COVID-19 [15]. The first version of the API was released on March 20, 2020 (version 1.0), followed by updates on March 25, 2020 (version 2.0), April 7, 2020 (version 3.0), and May 7, 2020 (version 4.0).

The app has been considered final from version 3.0; the set of risk factors and symptoms have reached their final form. However, the core logic of the interview, such as the flow of the interview, types of acquired data, and types of output recommendations, has been consistent from the first released version. In this study, we only considered interviews in the period between April 7 and August 6, 2020.

### Medical Foundation

The CRA tool’s logic was built around WHO guidelines [15] and WHO daily transmission reports [16]. The interview was designed to gather enough data to establish whether the user falls into any of the 3 categories mentioned in said guidelines as “Suspected case” for COVID-19; therefore, the reported symptoms may have resulted from SARS-CoV-2 infection.

For this reason, the interview consisted of 3 sets of questions that could be grouped into 3 categories:

- Risk factors and symptoms
- Places of residence and travel
- Contact with possible COVID-19 cases

In some cases, when this information was unnecessary to make a diagnosis, some questions were omitted.

### Data Analysis

The majority of the data were compared and presented with the use of descriptive statistics. Inferential statistics had to be omitted because of the significant differences in both compared populations and vastly different sample sizes. We decided to only use statistical analysis to compare comorbidities related to COVID-19 in both CRA and admission room groups. In CRA, *P* values were calculated with the test of proportions and in the admission room, with the Fisher exact test.

### Screen Deep Dive

The interview consisted of up to 8 consecutive screens. Not every screen had to be included; this is the maximum number of screens that the user could have been exposed to. If the patient reported emergency evidence (ie, acute dyspnea), the interview was terminated with an instruction to call an ambulance. The screens in the display order were “Welcome & Terms of Service,” Age and Sex Selection,” “Risk Factors,” “Symptoms,” “Red Flags,” “Possible Exposure to COVID-19,” “Travel and Residency,” and “Outcome.”

Nine risk factors were included to inquire about the user’s chronic illnesses and overall medical condition: diseases or drugs that weaken the immune system, obesity, long-term stay at a care facility or nursing home, diabetes, cancer, cardiovascular disease, history of chronic lung disease, history of chronic liver disease, and history of chronic kidney disease.

Some of these comorbidities have been described as negatively impacting COVID-19 infection outcomes [17]. We also included risk factors described in the Pneumonia Severity Index (PSI) as a negative prognostic factor indicating the need for hospitalization [18].

The symptom screens were oriented on inquiring about users’ symptoms that should raise clinical suspicion for COVID-19 according to WHO guidelines [15]. There was a list of 11 symptoms users could choose from: fever, cough, shortness of breath, fatigue, muscle pain, chills, headache, diarrhea, nausea, sore throat, and impaired taste or smell.

Furthermore, the interview focused on assessing red flags—immediate health threats to the user that should yield in cessation of the interview. To do so, the user was asked about rapid symptom deterioration, tachypnea, or hemoptysis.

There were 6 possible outcomes of the interview, which referred to the possibility of COVID-19 infection and the severity of symptoms:

- COVID-19 suspected, serious: “Call the emergency number. Avoid all contact.”
- COVID-19 suspected, nonserious: “Consult your health care provider. Avoid all contact.”
- Contact with COVID-19, no symptoms: “Quarantine.”
- Non-COVID-19, serious: “Call a doctor.”
- Non-COVID-19, nonserious: “Stay home and monitor your symptoms.”
- Asymptomatic: “Follow preventive measures.”

The extensive screen description and decision tree logic can be browsed in Multimedia Appendix 1.

### Comparison Group: Admission Room Analysis

To compare individuals completing the survey with real patients diagnosed with COVID-19 by health care professionals, we turned to the Infectious Diseases Admission Room of J. Gromkowski Hospital in Wrocław. We analyzed 291 cases of patients visiting the admission room between April 7 and August 6, 2020. All the patients reporting to the admission room were suspected of COVID-19 infection; no other cases of infectious diseases were consulted in the admission room at that time. They may have been brought to the admission room by ambulance, referred by the primary care physician, or admitted by themselves. We excluded patients below 18 years of age.

Each patient was interviewed and examined by the physician working in the admission room. The interview consisted of fixed elements, such as current symptoms, comorbidities, medication, history of travel, contact with COVID-19-positive persons, and workplace and family interview. Blood analysis, chest X-rays, and COVID-19 swabs were obtained in most cases.

The patient's history and examination, along with the additional tests, allowed them to decide on admission to the hospital or discharge. After 24 hours, the results of the COVID-19 genetic test (reverse transcription polymerase chain reaction [RT-PCR] from nasopharyngeal or pharyngeal swabs) were available, which allowed reaching the final diagnosis.

#### Setting

J. Gromkowski Hospital in Wrocław, Lower Silesian Voivodeship, Poland, is 1 of the specialist hospitals in that city. There are 2 infectious disease wards in the hospital. The Infectious Diseases Admission Room serves as the place for preliminary triage, diagnosis, and treatment of incoming patients suspected of contracting infectious diseases. During the COVID-19 pandemic, it served as the main consultation facility of COVID-19-suspected cases.

#### Population

In this study, we analyzed the Infectious Diseases Admission Room cases between April 7 and August 6, 2020. We focused on the set of reported symptoms, comorbidities, contact with COVID-19 cases, and travel history. Our goal was to determine the patient profile, meaning assessing the set of symptoms connected with COVID-19 cases compared to non-COVID-19 cases.

Finally, we wanted to compare the sociodemographic and clinical characteristics of hospital patients and the ones completing the self-assessment interview.

#### Symptoms

In the study, we screened for 8 symptoms that are suggestive of COVID-19 infection: cough, fever, dyspnea, diarrhea, myalgia, rhinorrhea, taste and smell abnormalities, and pharyngeal pain.

## Results

### Demographics and Groups Characteristics

#### CRA Tool

**Figure 1:**
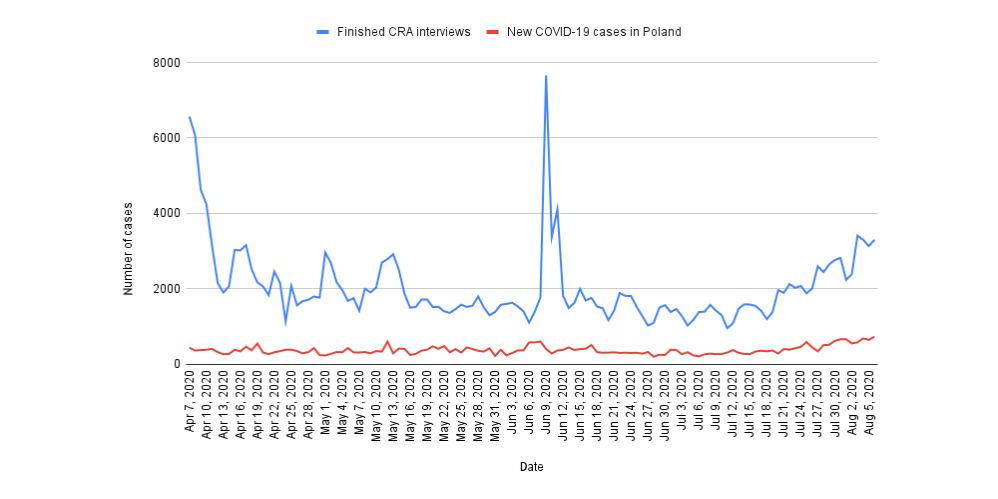
Finished CRA interviews daily (blue line); for comparison, daily number of new diagnosed COVID-19 cases in Poland (red line). CRA: COVID-19 risk assessment.

Of the 697,903 individual interviews performed on the CRA tool between April 7 and August 6, 2020, a total of 248,862 (35.66%) individual interviews met the inclusion criteria. Most of these interviews came from the government portal of the Polish Ministry of Health, which embedded the app within its website [19]: 117,311 (47.14%) of all interviews. In addition, 91,805 (36.89%) interviews were performed on the original CRA website [20], and 17,767 (7.14%) interviews were performed on the COVID-19 mobile app commissioned by the Polish Ministry of Health. Other notable institutions adopting the CRA tool and providing us interviews analyzed in the study included PZU Zdrowie (Polish biggest private health care provider), Dovera (private health care provider in Slovakia), Global Excel (medical assistance company operating in the U.S. and Canada), and others [21]. The CRA tool is offered in 37 languages in total: Polish, English, Slovak, Ukrainian, Portuguese-Brazilian, and Russian are the most popular languages. However, only Polish-speaking users met the inclusion criteria (Figure 1).

**Figure 2:**
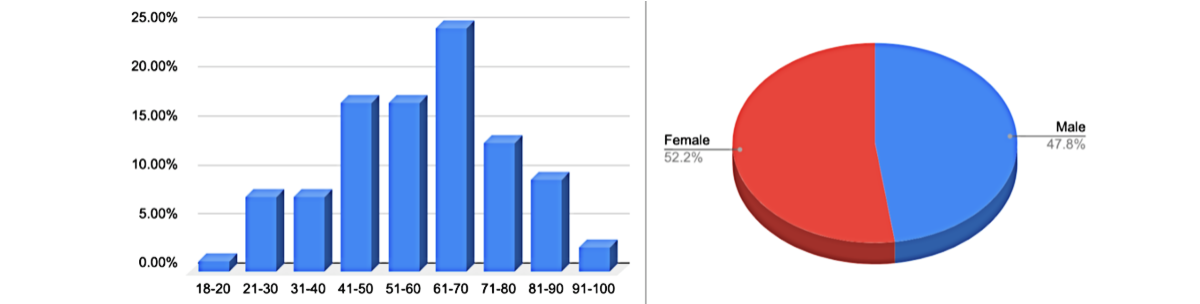
Age and sex distribution of admission room patients (N=291).

Most of the respondents were between 18 and 40 years old (n=158,998 [63.89%] of all respondents). The least prevalent were users between 80 and 90 years old (n=498, 0.2%). The mean age was 37 years. The study included 130,966 (52.63%) males and 117,896 (47.37%) females (Figure 2).

#### Admission Room

**Figure 3:**
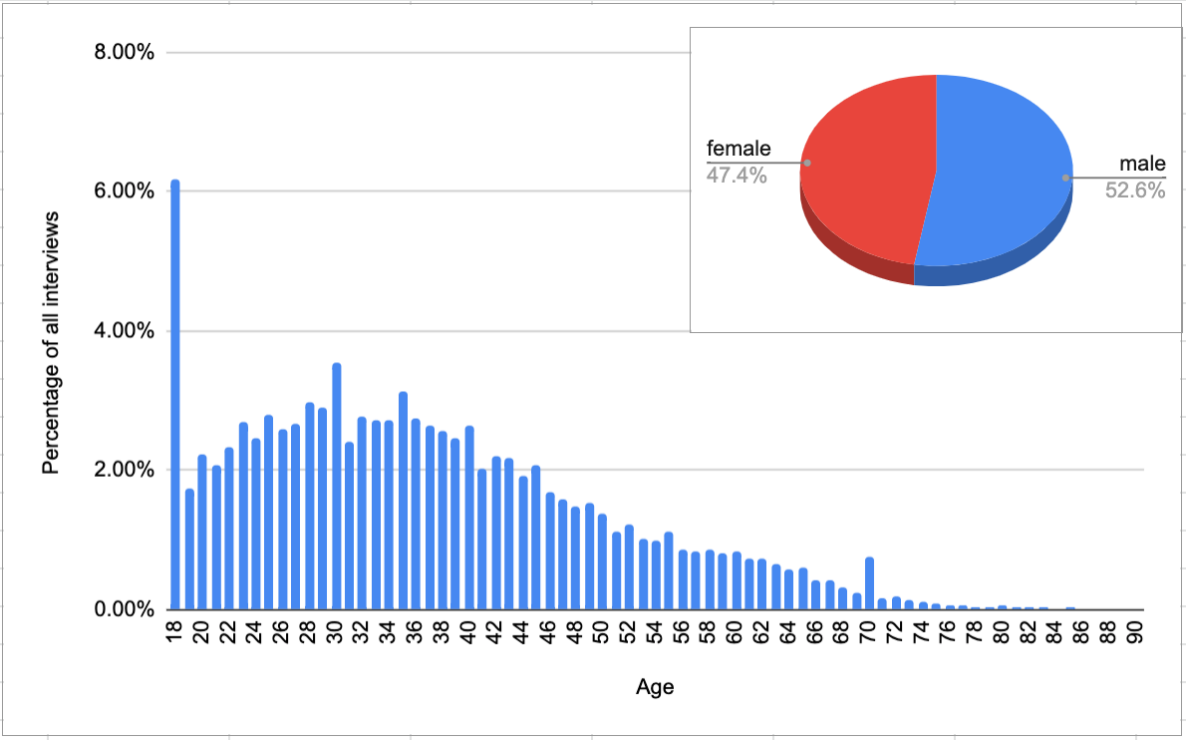
Age and sex distribution of CRA users. CRA: COVID-19 risk assessment.

The study included 291 patients who visited the Infectious Diseases Admission Room of J. Gromkowski Hospital in Wrocław between April 7 and August 6, 2020. There were 152 (52.23%) women and 139 (47.77%) men enrolled in the study. Most of the patients were between 41 and 70 years old. The mean age was 58 years; the median age was 60 years (Figure 3).

### Outcomes and Triage Results

#### CRA Tool

Among the users of the CRA tool, the most common interview result was “asymptomatic” or “Follow preventive measures,” which was displayed to 98,081 (39.41%) of the 248,862 users. This subgroup consisted of users who answered the questionnaire but denied having any symptoms or COVID-19 exposure.

The second-most common triage outcome was “non-COVID-19, nonserious” or “Stay home and monitor your symptoms” for 73,145 (29.39%) of the 248,862 users. This subgroup comprised users who answered the questionnaire and reported only mild symptoms, such as fatigue, muscle pain, chills, headache, diarrhea, nausea, sore throat, and impaired taste or smell, but denied having any COVID-19 exposure (contact or travel). These users were not suspected of SARS-COV-2 infection according to the diagnosing rules proposed by WHO at that time [15].

Both these groups added up to 171,226 (68.80%), which made them the majority of the CRA tool users. See Tables 1 and 2 for details of the CRA tool group.

The third-most common triage outcome was “Call the emergency number,” which was recommended to 30,494 (12.25%) of the users. These were referred to as “COVID-19 suspected, serious” cases. Users who received that recommendation reported red-flag symptoms indicating respiratory distress or potentially severe infection (shortness of breath in the elderly, tachypnea, hemoptysis, high-grade fever, rapid symptom deterioration) and confirmed potential COVID-19 exposure.

Of the 248,862 users, 21,980 (8.83%) were classified as “Non-COVID-19, serious”: they received a “Call a doctor” recommendation. These users were not suspected of SAR-CoV-2 infection, because they had not met the WHO criteria of the suspected case at the time [15], but were advised to obtain a teleconsultation due to potentially severe symptoms: shortness of breath, high-grade fever, and fever and cough in the elderly.

The least prevalent group was the “COVID-19 suspected, nonserious” or “Consult your health care provider. Avoid all contact,” displayed to 9513 (3.82%) users. This group reported symptoms and COVID-19 exposure suggestive of SARS-CoV-2 infection but denied having potentially serious symptoms calling for an in-person consultation. They were advised to self-isolate and undergo a COVID-19 test.

**Table 1:** Distribution of CRA^a^ interview outcomes (N=248,862).

Triage	Patients, n (%)
Asymptomatic	98,081 (39.41)
Non-COVID-19, nonserious	73,145 (29.39)
COVID-19 suspected, serious	30,494 (12.25)
Non-COVID-19, serious	21,980 (8.83)
Quarantine	15,649 (6.29)
COVID-19 suspected, nonserious	9513 (3.82)

^a^CRA: COVID-19 risk assessment.

**Table 2:** Distribution of CRA^a^ interview outcomes: matrix of the clinical suspicion of COVID-19 (N=248,862).

Severity of presented symptoms	COVID-19 suspected, n (%)	Non-COVID-19, n (%)
Serious	30,494 (12.25)	21,980 (8.83)
Nonserious	9513 (3.82)	171,226 (68.80)

^a^CRA: COVID-19 risk assessment.

#### Admission Room

Of the 291 patients, 232 (79.73%) tested positive for COVID-19 and 59 (20.27%) tested negative for COVID-19. Of the 152 women, 126 (82.89%) were COVID-19 positive and 26 (17.11%) were COVID-19 negative. Of the 139 men, 106 (76.26%) were COVID-19 positive and 33 (23.74%) were COVID-19 negative.

Most of the patients (n=167, 57.39%) of the admission room group were classified by consulting physicians as patients in good general condition, 85 (29.21%) of the patients were judged to be in moderate general condition, 30 (10.31%) were in a bad general condition, and 9 (3.09%) were in a severely bad general condition.

### Comorbidities

The number of reported comorbidities in the self-assessment app was 71,515; at least 1 risk factor was reported in 71,523 (28.74%) of the interviews. In other words, in 177,339 (71.26%) of the interviews, users did not report any comorbidity.

The most frequently reported comorbidity in both the CRA tool users and the admission room patients was cardiovascular disease, defined as hypertension, coronary disease, or heart insufficiency and confirmed by 37,628 (15.12%) of 248,862 CRA tool users and 138 (47.42%) of 291 admission room patients.

The distribution of other comorbidities shaped quite similarly between the 2 compared groups:

- In the CRA tool group, the other common risk factors were chronic lung disease (8337/248,862, 3.35%) and diabetes (5998/248,862, 2.41%).
- In the admission room group, the other common risk factors were diabetes (56/291, 19.24%), cancer (active neoplasms of all types, including of hematological origin; 30/291, 10.31%), and chronic lung disease (22/291, 7.56%).

A relatively high percentage of people reporting immunosuppression in the CRA tool group (weakened immune system; 14,708/248,862 [5.91%] of users) compared to the admission room group (6/291, 2.06%) suggests this risk factor might have been misinterpreted and misused despite the extensive description explaining the nature and examples of immunosuppression (available in Multimedia Appendix 2).

In general, admission room patients more often were burdened with comorbidities compared to CRA tool users. This can be explained by a higher average age of admission room patients compared to CRA tool users (Table 3).

**Table 3:** Distribution of comorbidities^a^ in the CRA^b^ tool and admission room groups.

Comorbidities	CRA tool				Admission room		
	COVID-19 positive (N=40,007), n (%)	COVID-19 negative (N=193,206), n (%)	*P* value^c^	COVID-19 positive (N=232), n (%)		COVID-19 negative (N=59), n (%)	*P* value^d^
Cardiovascular diseases	9346 (23.36)	26,296 (13.61)	<.001	125 (53.88)		13 (22.03)	<.001
Diabetes	1680 (4.20)	4012 (2.08)	<.001	51 (21.98)		5 (8.47)	.02
Current cancer	818 (2.04)	1517 (0.79)	<.001	28 (12.07)		2 (3.39)	.06
Diagnosed chronic lung disease	2461 (6.15)	5425 (2.81)	<.001	20 (8.62)		2 (3.39)	.27
History of chronic liver disease	1064 (2.66)	2140 (1.11)	<.001	7 (3.02)		1 (1.69)	.99
History of chronic kidney disease	967 (2.42)	1851 (0.96)	<.001	7 (3.02)		1 (1.69)	.99
Weakened immune system	4309 (10.77)	9629 (4.98)	<.001	5 (2.16)		0	0.59

^a^Overall comorbidities: There were 20,645 comorbidities in COVID-19 positives and 50,870 comorbidities in COVID-19 negatives in the CRA tool group. There were 243 comorbidities in COVID-19 positives and 24 comorbidities in COVID-19 negatives in the admission room group.

^b^CRA: COVID-19 risk assessment.

^c^*P* values for CRA: test of proportions.

^d^*P* values for the admission room: Fisher exact test.

### Symptom Distribution

Overall, the most commonly reported symptoms differed between the CRA tool and the admission room groups. CRA interviews were dominated by mild symptoms, such as fatigue (61,544/248,862, 24.73%), cough (54,575/248,862, 21.93%), and headache (45,417/248,862, 18.25%). Meanwhile, the admission room patients presented with more serious symptoms, such as fever (175/291, 60.14%), cough (168/291, 57.73%), shortness of breath (114/291, 39.18%), and fatigue and muscle pain (59/291, 20.27% for both).

In the admission room group, the distribution of the most common symptoms among COVID-19-positive (232/291, 79.73%) and COVID-19-negative (59/291, 20.27%) patients was fairly similar: fever (n=146 [62.9%] of COVID-19 positives, n=29 [49.2%] of COVID-19 negatives), cough (n=138 [59.5%] of COVID-19 positives, n=30 [50.8%] of COVID-19 negatives), and shortness of breath (n=87 [37.5%] of COVID-19 positives, n=27 [45.8%] of COVID-19 negatives).

In contrast, the presentation of the COVID-19-suspected and COVID-19-nonsuspected individuals differed substantially. COVID-19-suspected users commonly reported symptoms such as fever, cough, and shortness of breath, while COVID-19-nonsuspected users commonly reported headache, cough, and fatigue. For details see Table 4.

**Table 4:** Symptom and risk factor distribution of CRA^a^ tool users and admission room patients.

Symptom or risk factor	CRA tool			Admission room	
	COVID-19 positive (N=40,007), n (%)	COVID-19 negative (N=193,206), n (%)	COVID-19 positive (N=232), n (%)		COVID-19 negative (N=59), n (%)
Cough	25,062 (62.64)	29,521 (15.28)	138 (59.48)		30 (50.85)
Fever	23,123 (57.80)	20,292 (10.50)	146 (62.93)		29 (49.15)
Symptoms getting worse quickly	19,816 (49.53)	0	N/A^b^		N/A
Shortness of breath	15,157 (37.89)	12,717 (6.58)	87 (37.50)		27 (45.76)
Faster breathing	12,964 (32.40)	0	N/A		N/A
Fatigue	5987 (14.96)	52,630 (27.24)	40 (17.24)		19 (32.20)
Headache	4497 (11.24)	38,115 (19.73)	19 (8.19)		4 (6.78)
Sore throat	3975 (9.94)	35,645 (18.45)	17 (7.33)		9 (15.25)
Muscle pain	3351 (8.38)	27,015 (13.98)	42 (18.10)		17 (28.81)
Coughing up blood	2006 (5.01)	0	1 (0.43)		0
Chills	1906 (4.76)	13,740 (7.11)	2 (0.86)		2 (3.39)
Diarrhea	1242 (3.10)	14,109 (7.30)	35 (15.09)		4 (6.78)
Contact with infected person	1005 (2.51)	0	166 (71.55)		5 (8.47)
Nasal catarrh	954 (2.38)	6134 (3.17)	20 (8.62)		5 (8.47)
Loss of smell or taste	947 (2.37)	6034 (3.12)	39 (16.81)		2 (3.39)
Nausea	911 (2.28)	10,599 (5.49)	10 (4.31)		3 (5.08)
No contact with infected person	0	193,206 (100)	66 (28.45)		54 (91.53)

^a^CRA: COVID-19 risk assessment.

^b^N/A: not applicable.

### Comparative Results

Fever and cough were the most commonly reported symptoms of COVID-19 in CRA tool users and admission room patients: fever occurred in 23,123/40,007 (57.80%) and 146/232 (62.93%) of the studied groups, respectively, while cough occurred in 25,062/40,007 (62.64%) and 138/232 (59.48%) of the studied groups, respectively. Pneumonia, characterized as the presence of fever, cough, and dyspnea, has been proven to be the most prevalent clinical presentation of COVID-19 in many studies [22-25].

Cardiovascular disease and diabetes occurred significantly more commonly in the COVID-19-positive than in the COVID-19-negative group both in the CRA tool (9346/40,007 [23.36%] vs 26,296/193,206 [13.61%] for cardiovascular disease, *P*<.001; 1680/40,007 [4.20%] vs 4012/40,007 [2.08%], *P*<.001 for diabetes) and in the admission room (125/232 [53.88%] vs 13/59 [22.03%] for cardiovascular disease; 51/232 [21.98%] vs 5/59 [8.47%], *P*<.001 for diabetes) group.

Anosmia or ageusia (2/59, 3.39%) occurred more frequently in the admission room group in COVID-19-positive than in COVID-19-negative patients. In the app, we did not observe a similar finding, probably due to the rapid cessation of the interview in high-triage scenarios.

Anosmia or ageusia occurred more frequently in mild than in severe COVID-19 in the CRA tool group (3849/40,007 [9.62%] vs 40/40,007 [0.10%]). This is consistent with studies suggesting that olfactory and gustatory disturbances are among the most commonly reported symptoms in mild-to-moderate COVID-19 [26].

The average age of users of the COVID-19 self-assessment app was 37 years, whereas the average age of admission room patients was 58 years.

Fatigue, chills, nausea, and sore throat did not turn out to be diagnostically relevant for diagnosing COVID-19. In both CRA tool and admission room groups, they occurred more frequently in non-COVID-19 individuals.

## Discussion

### Principal Findings

The CRA tool ceased to be supported on August 16, 2021. As of now, most of the COVID-19 diagnostics are run by the Infermedica artificial intelligence (AI) engine [27], and the CRA tool is supported only in selected use cases (ie, the Polish Ministry of Health) [19].

The CRA tool, as it served as a means of screening and self-education, did not substitute for consultations in the admission room for symptomatic users. The tool could not confirm or exclude SARS-CoV-2 infection, as it cannot perform a laboratory examination. Hence, it does not substitute for physicians' interactions. However, our tool exercised the purpose of remote triage. CRA did not overlook truly symptomatic cases; users with potentially worrisome symptoms, such as fever or shortness of breath, were identified and advised to obtain a consultation or schedule a COVID-19 test.

The compared groups—one that completed the online interview and one that reported to the hospital—differed in age distribution, the presence of risk factors, and probably the severity of symptoms reported. The difference between both groups impacted the results of the study, but it also showed some limitations of remote diagnostic tools, such as CRA—as patients potentially the most vulnerable to COVID-19 are also the least prevalent group accessing the internet for a health checkup. It is observed, however, that younger patients also suffer from COVID-19 infection, and with the next waves of pandemics, infections in young adults will become more prevalent [28]. This growing group of patients could have benefitted from remote triage assessment tools, such as CRA.

Taste and smell disorders occurred more commonly in the admission room group than in the CRA tool group (39/232 [16.81%] vs 947/40,007 [2.37%] for COVID-19-suspected individuals). In search of a possible explanation of this finding, we turned to the logic of WHO guidelines used in the CRA tool at that time. They did not distinguish smell and taste disorders as key diagnostic factors [15]. Once the importance of symptoms such as smell and taste disorders came to the attention of academics [29], WHO reflected these findings in the updated guidelines for suspecting COVID-19 infection (on August 7, 2020). WHO emphasized adjacent symptoms, such as diminished taste or smell, and reduced the significance of fever in suspecting COVID-19 infection. The newer versions of the CRA tool, not described in this paper, follow the guidelines, increasing their diagnostic importance.

It was not possible to assess the actual number of false-negative cases in the CRA tool due to a lack of data. However, we know that among the admission room records, 31 (13.78%) of 225 patients did not present with fever or dyspnea but still tested positive for COVID-19. These patients would have been classified as non-COVID-19 cases by the app.

Concomitant symptoms, such as fatigue, headache, and diarrhea, occurred infrequently in severe COVID-19-positive cases in the app. This may have been caused by the premature cessation of the interview for safety reasons.

The overall number of COVID-19-suspected cases in the CRA tool was 40,007 (16.08%) of 248,862 individual interviews. This number corresponds with the number of scheduled tests for novel coronavirus because in both these cases, we deal with the suspicion of COVID-19 based on presented history and symptoms. During a similar period, between May 11 and August 3, 2020, there were 17,864,205 tests for SARS-CoV-2 performed [30].

### Limitations of the Study

#### Possible Misinterpretation of Red-Flag Questions

The outcomes of the self-assessment triage tool highlighted room for improvement with regard to phrasing questions in web apps for the common user. The “symptoms getting worse quickly” red flag was meant to pinpoint a swiftly deteriorating user's general condition, which is a premise for hospitalization. However, a comparable number of confirmative and declined answers suggest that many of these answers could have been false positives. This answer might have been overly reported by the respondents, who may have misinterpreted its scope. In many cases, this occurrence may have led to the overtriage of urgent COVID-19 case recommendation (“Call the emergency number.”).

#### Bias of the Sample

As the tool was publicly available to everyone and no check-in or login was required, there is a possibility that some users did not present the symptoms they reported and used the tool only out of curiosity or for educational purposes. However, this bias is probably limited by the size of the group tested with the self-assessment tool.

#### More Detailed Screening in the Admission Room Sample

Screening in the admission room is always more exhaustive than in any self-assessment tool. There are a couple of contributing factors:

- Physical examinations cannot be substituted by any questions asked by the symptom checker.
- A general appearance provides valuable clinical information to experienced clinicians.
- There is a closed set of symptoms to choose from in the CRA tool.
- After detecting a potential red flag, the tool is designed to terminate the interview without inquiring about concomitant symptoms.

### Conclusions

Comparing the symptomatology of users interacting with the CRA tool and those visiting the admission room revealed 2 major patient groups that could have benefited from implementing the self-assessment app in preclinical triage settings.

The first group were patients with typical COVID-19 symptoms: cough and fever, sometimes accompanied by shortness of breath, tachypnea, fatigue, headache, and muscle pain. Some of these patients had additional comorbidities, such as diabetes or cardiovascular disease, that could have impacted the clinical course of COVID-19 [17]. The CRA tool could recognize patients with such symptoms as potentially COVID-19 positive and directed them to the proper care. The CRA tool was accurate in identifying patients at risk: every patient reporting a potential red-flag symptom, such as rapid symptom deterioration or acute dyspnea and tachypnea, was advised to seek immediate medical attention in the emergency room or was instructed to call the ambulance.

The other group were patients with no symptoms suggesting COVID-19 infection but still searching for answers as to whether they could be infected and what they should do. Oligosymptomatic and asymptomatic users, who constituted the majority of individuals interacting with the tool, were educated about their symptoms and advised to refer to the primary care in the case of symptom worsening. CRA has played an educational role in advising on isolation precautions, organizing quarantine, and referring for further reading using evidence-based sources, such as WHO and the CDC.

It seems that these types of solutions may serve as health information hubs for oligosymptomatic individuals and means of remote triage for a vast audience. They possess the ability to identify patients at risk, providing them with next-step recommendations, as well as sieving out asymptomatic individuals, providing them with evidence-based education materials. Such patients were the most prevalent (171,226 [68.80%] of the 248,862 CRA tool users).

As the study did not examine the intention of the user, it is uncertain what portion of such patients would visit a health care professional unnecessarily; further studies are required to assess the exact impact of online tools on reducing unnecessary visits. Still, as we observed oligosymptomatic patients visiting the hospital admission room, it can be assumed that some portion of such visits could be prevented by providing reassuring information to the patient through the online tool.

## Appendix

Multimedia Appendix 1 Extensive screen description and decision tree logic.

Multimedia Appendix 2 Distribution of symptoms and comorbidities in the CRA tool, displayed by the triage outcome. CRA: COVID-19 risk assessment.

## Abbreviations

API: Application Program Interface
CDC: Centers for Disease Control and Prevention
CRA: COVID-19 risk assessment
WHO: World Health Organization
