# Supplementary material for: Application of a Web-based Self-assessment Triage Tool During the COVID-19 Pandemic: Descriptive Study
Source: JMIR Hum Factors. 2022 Apr 4;9(2):e34134. doi: 10.2196/34134 (PMC8982648; doi:10.2196/34134)
Supplement: Multimedia Appendix 1 [file humanfactors_v9i2e34134_app1.docx]

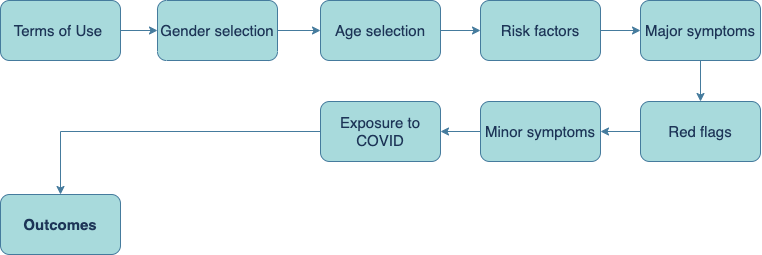


*1. Overview of the flow of the interview*


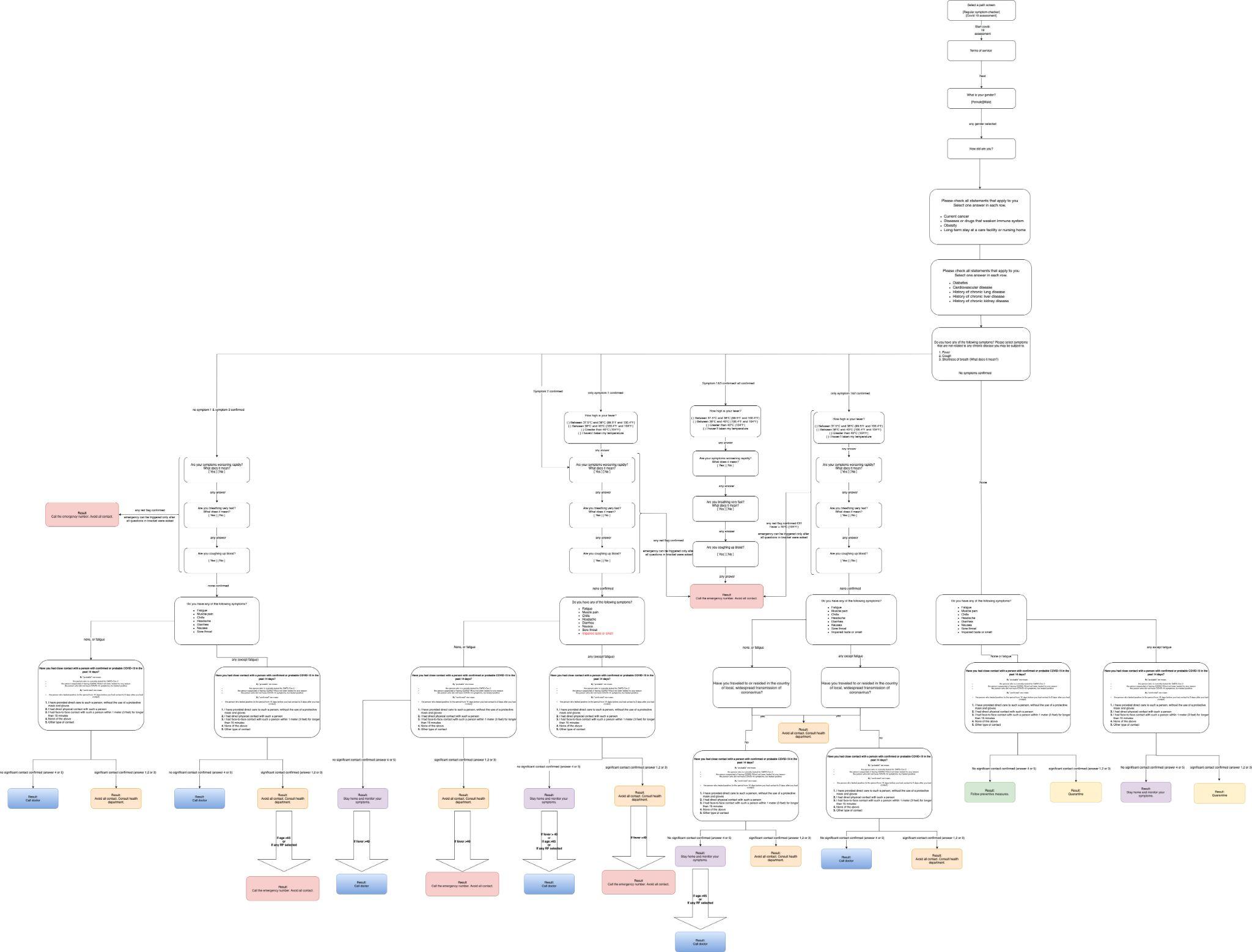


*2. The overview of the decision tree logic. Detailed browsing available at* [Infermedica COVID-19 Risk Assessment flow.jpg](https://drive.google.com/file/d/1ClkVr6cRUh0nolZnrSvv4uidIsbs-XrI/view?usp=sharing)

*
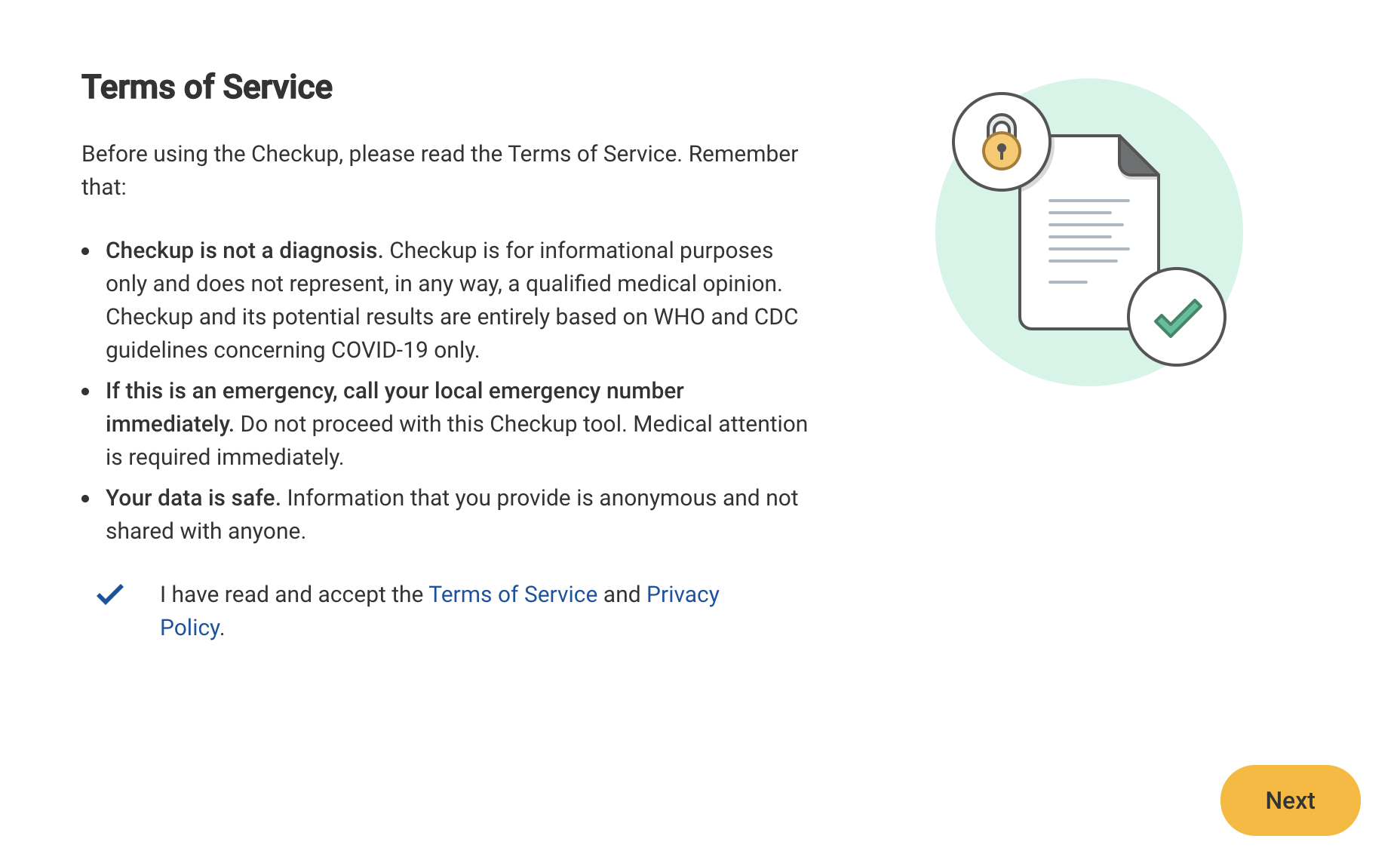
*

*3a. Screenshots of all questions provided in the tool- Terms of Service*

*
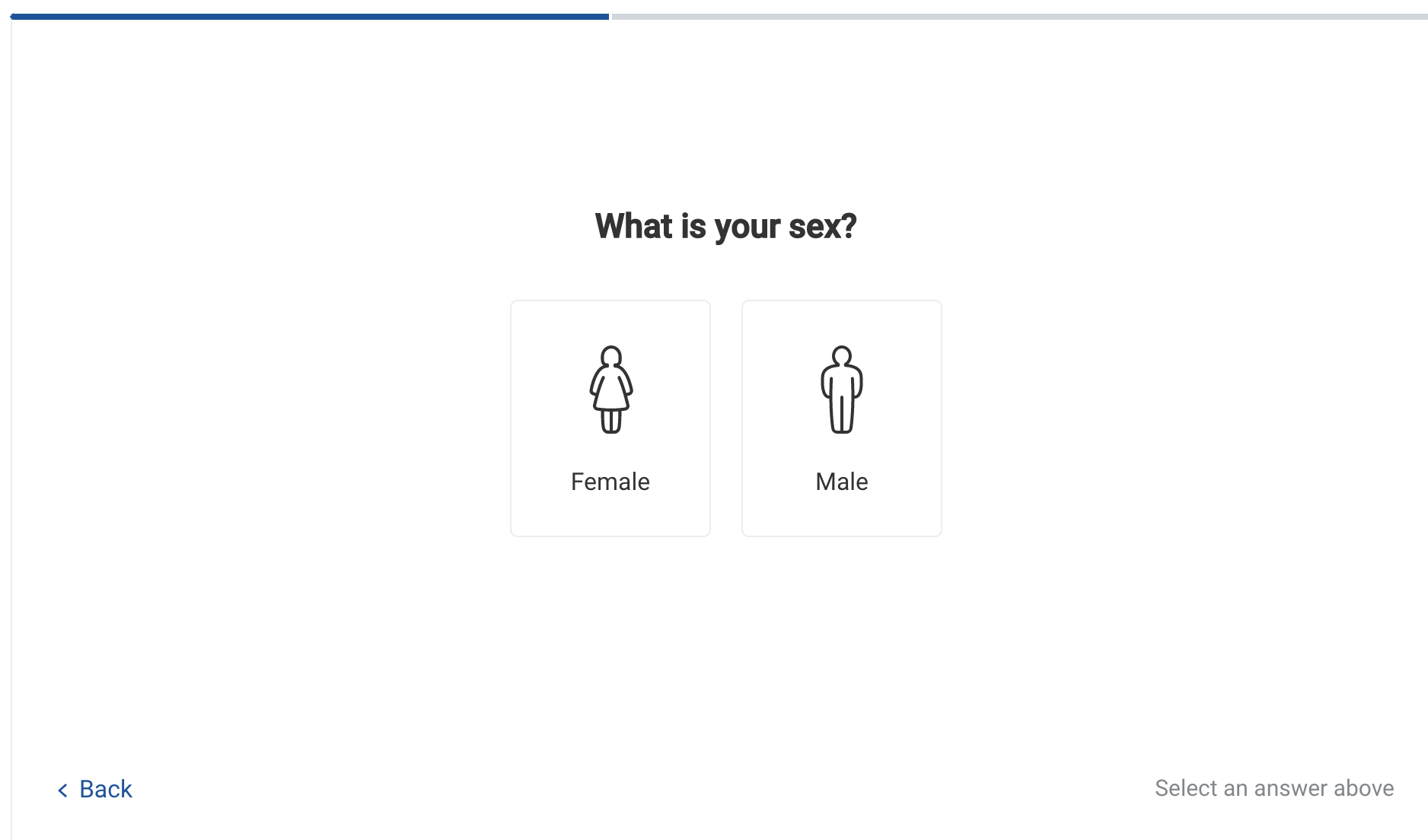
*

*3b. Screenshots of all questions provided in the tool- gender selection screen*

*
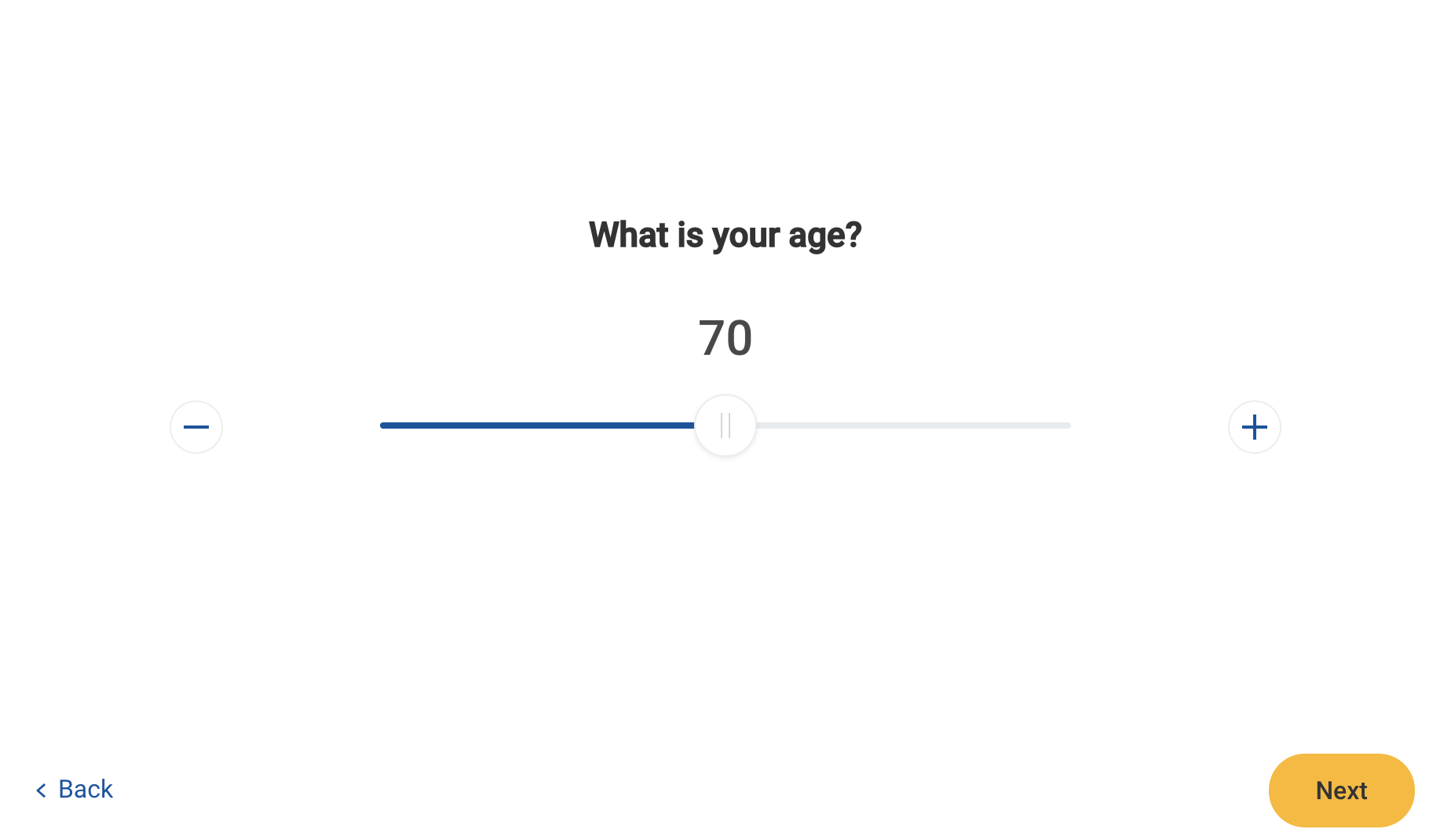
*

*3c. Screenshots of all questions provided in the tool- Age selection screen*

*
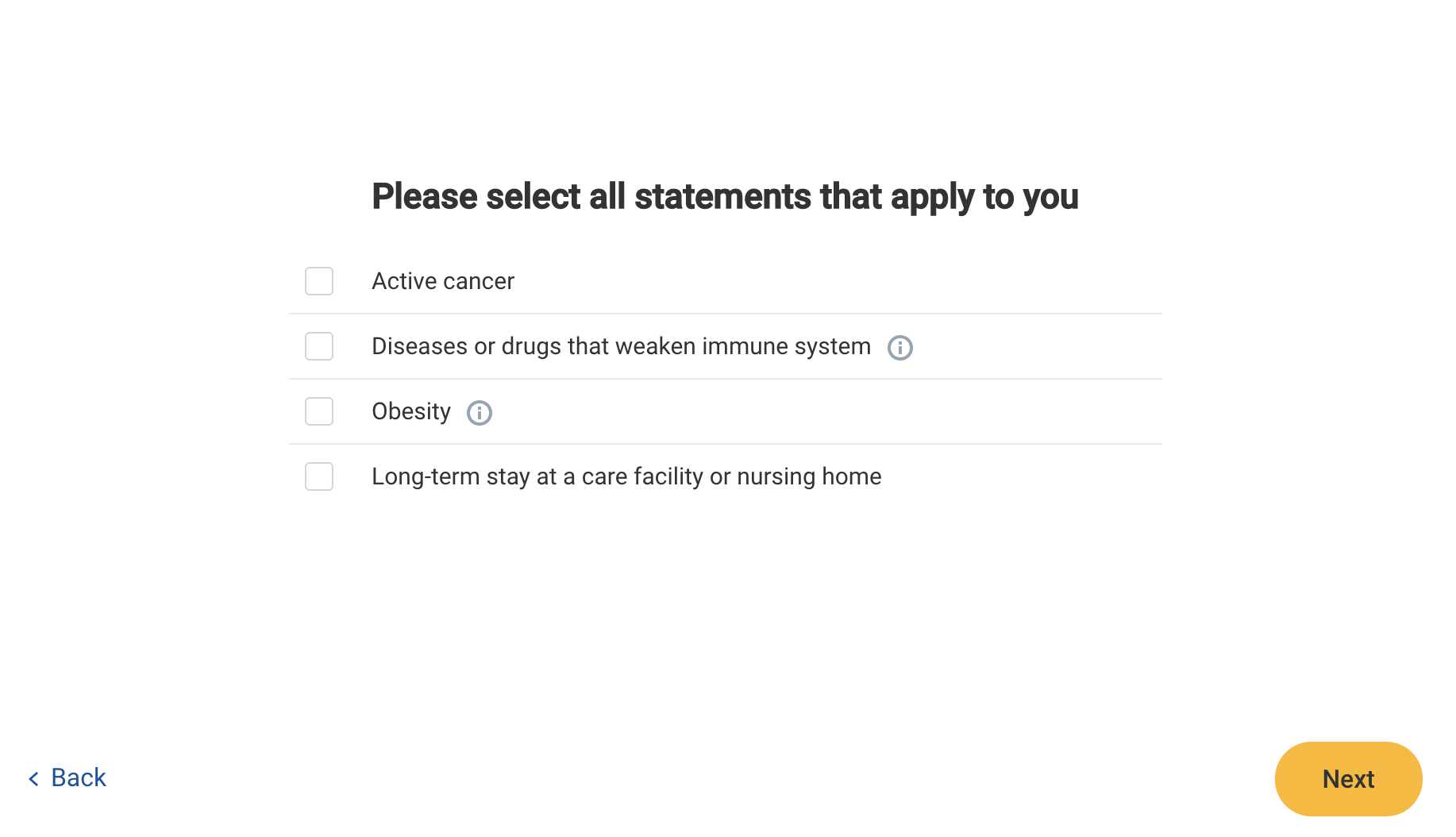
*

*3d. Screenshots of all questions provided in the tool- 1st risk factor screen*

*
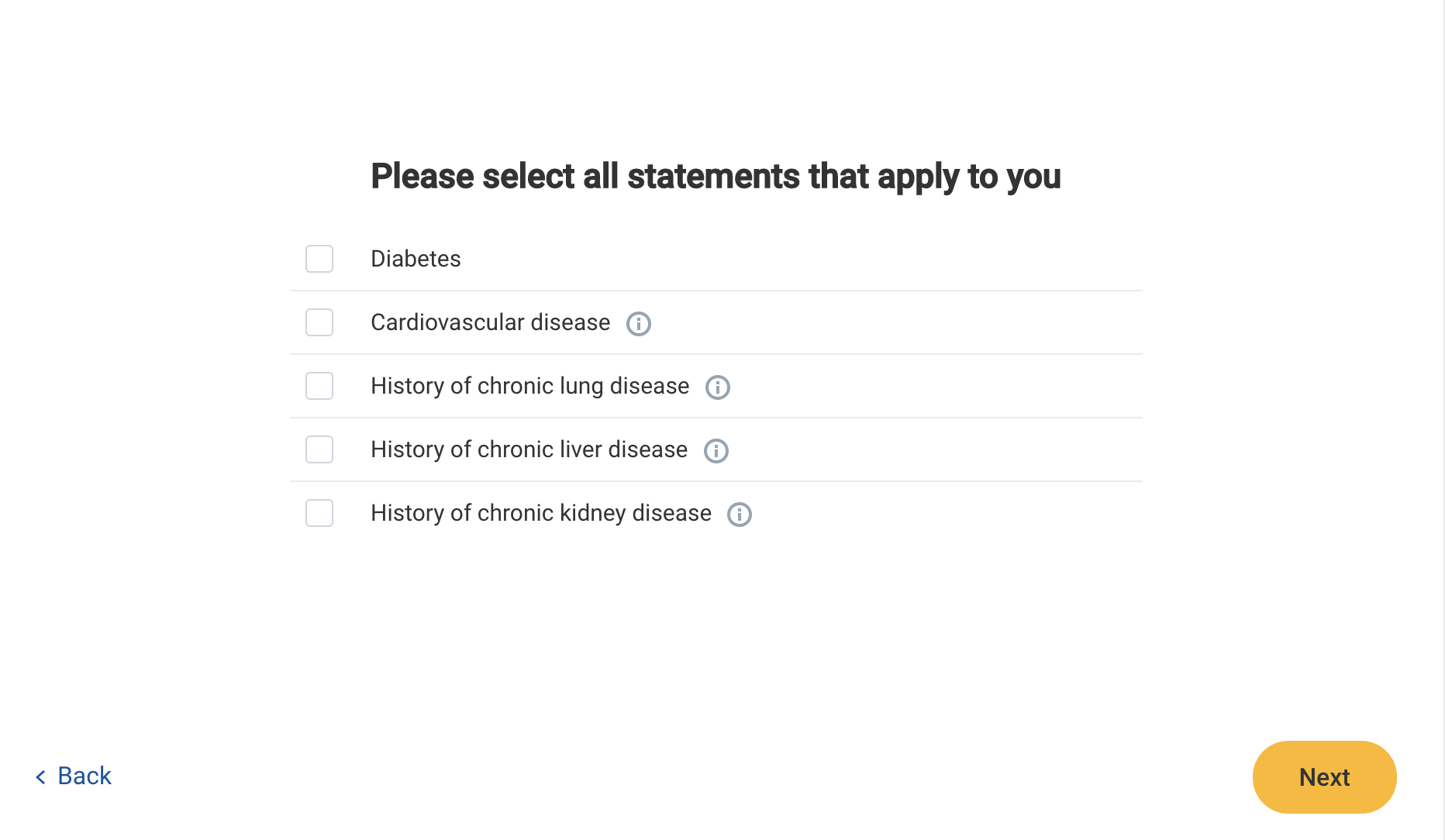
*

*3e. Screenshots of all questions provided in the tool- 2nd risk factor screen*

*
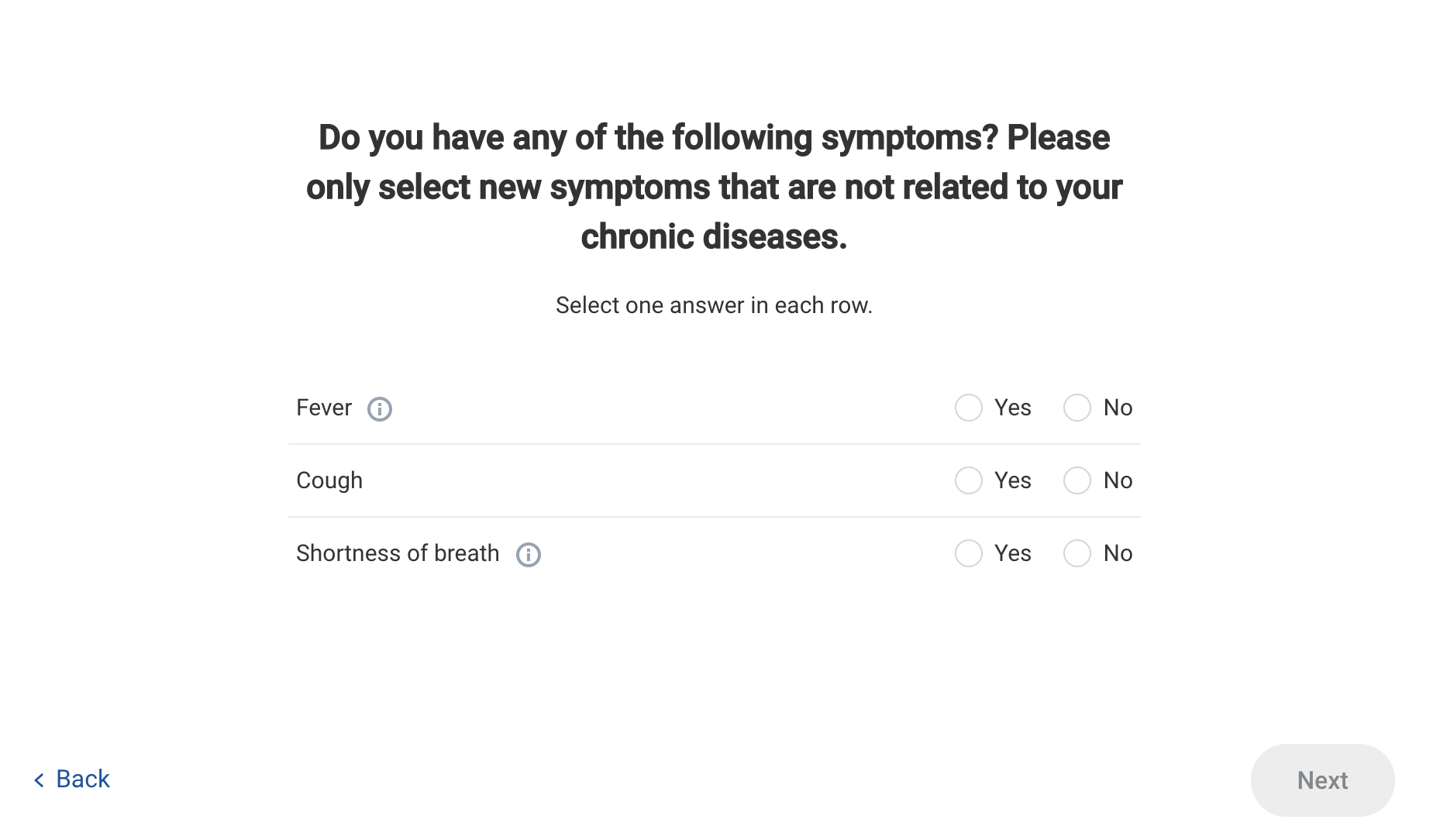
*

*3f. Screenshots of all questions provided in the tool- Main symptoms screen*

*
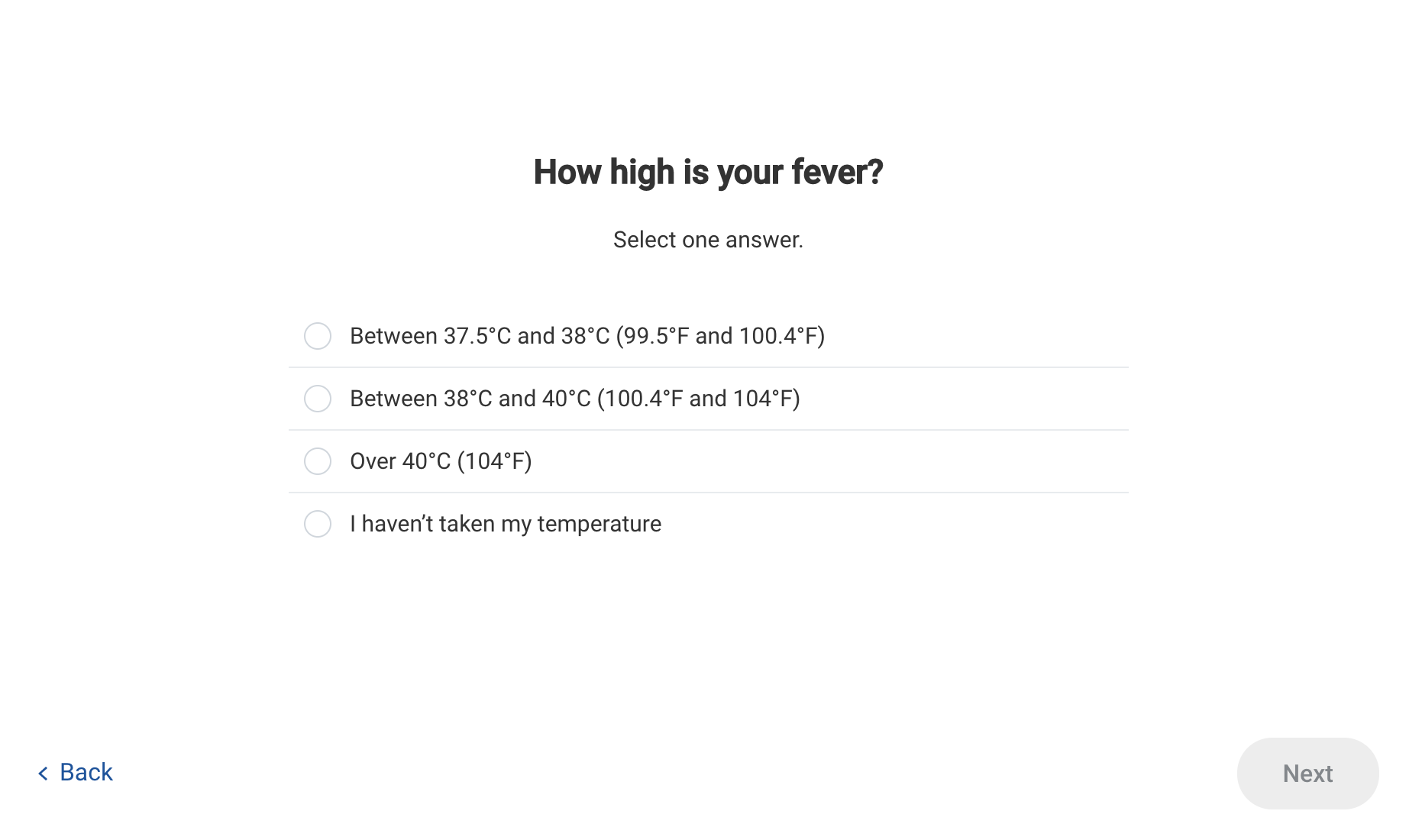
*

*3g. Screenshots of all questions provided in the tool- choosing degree of fever*

*
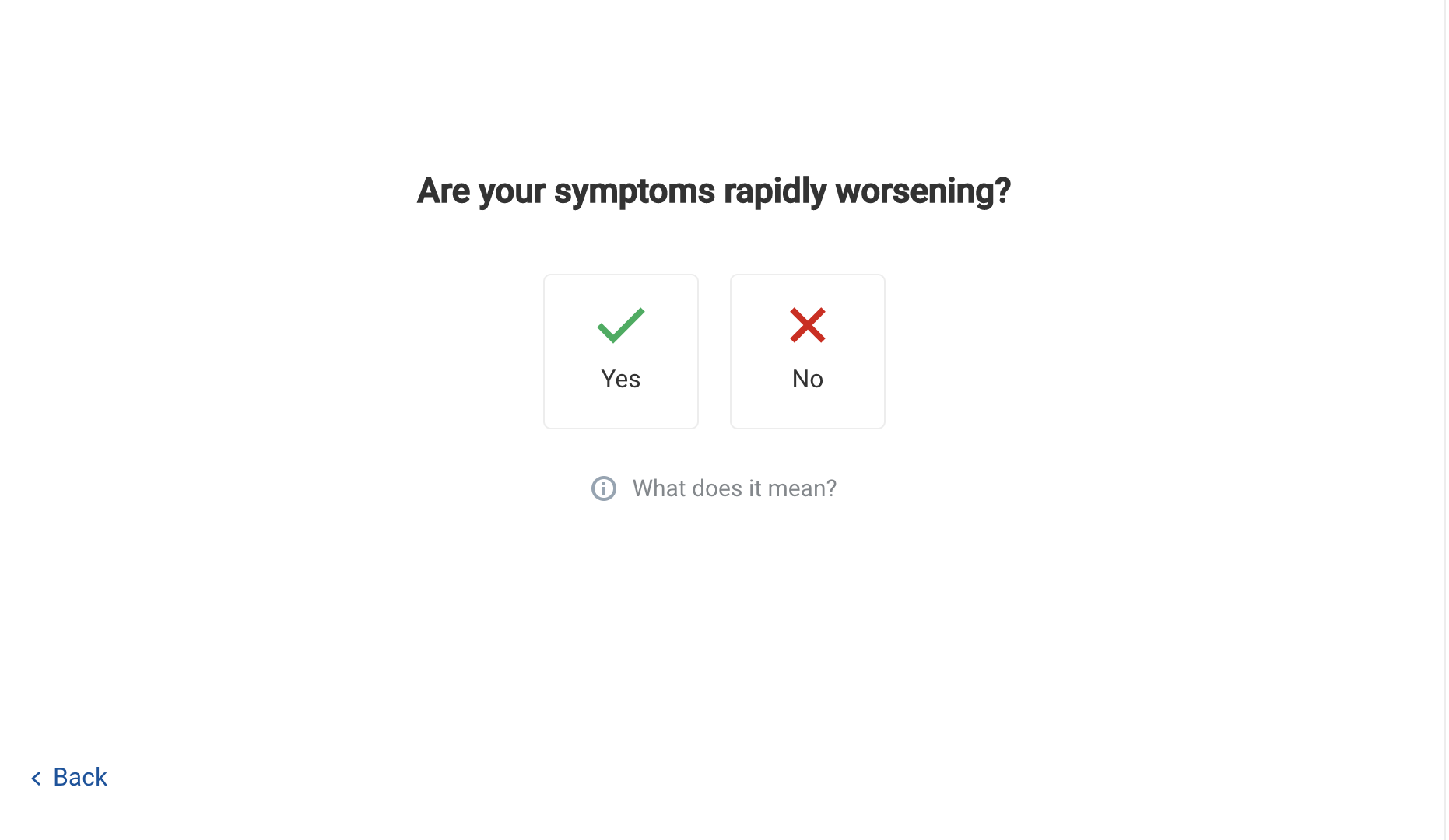
*

*3h. Screenshots of all questions provided in the tool- Rapid symptoms worsening*

*
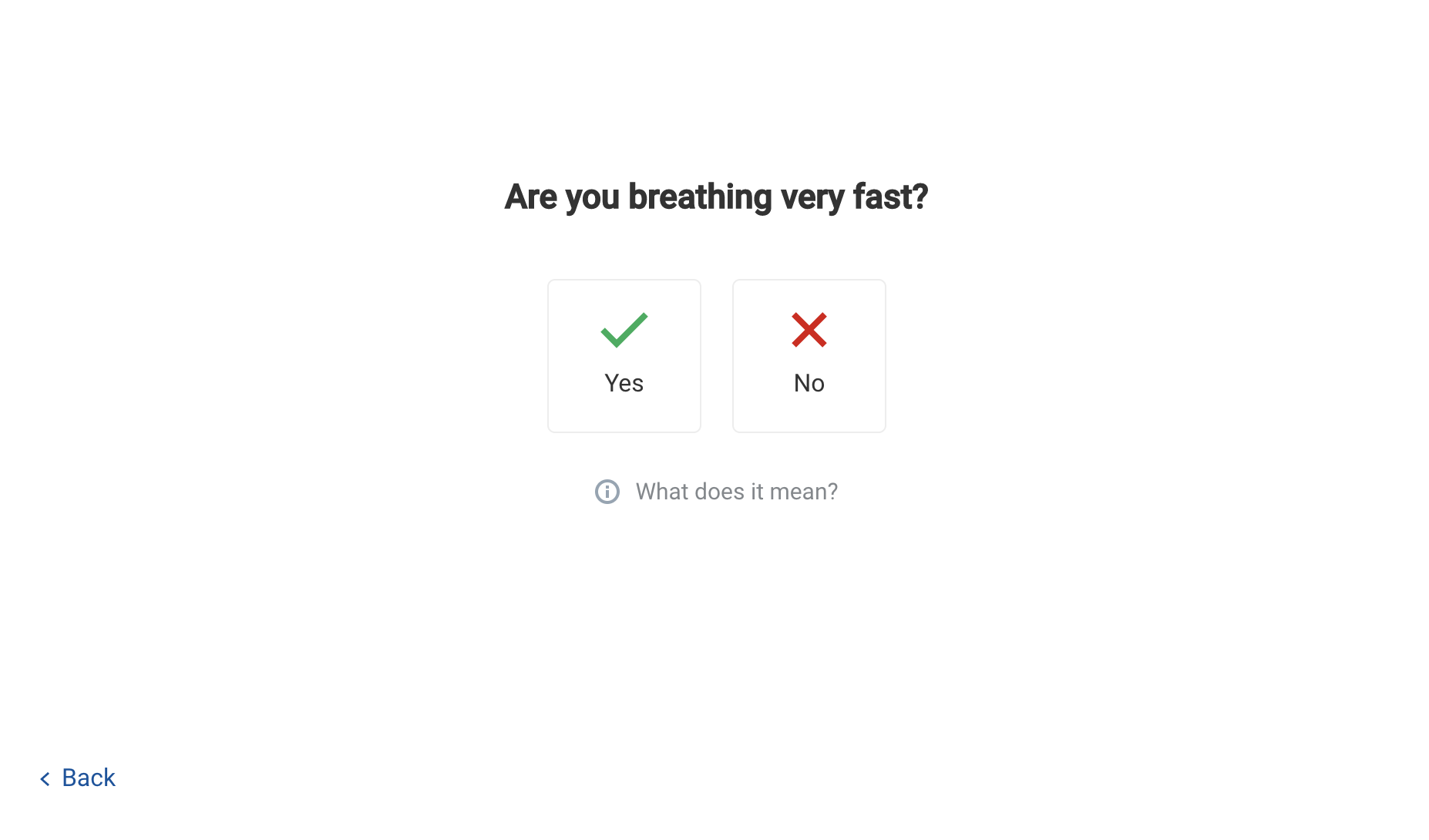
*

*3i. Screenshots of all questions provided in the tool- Tachypnea*

*
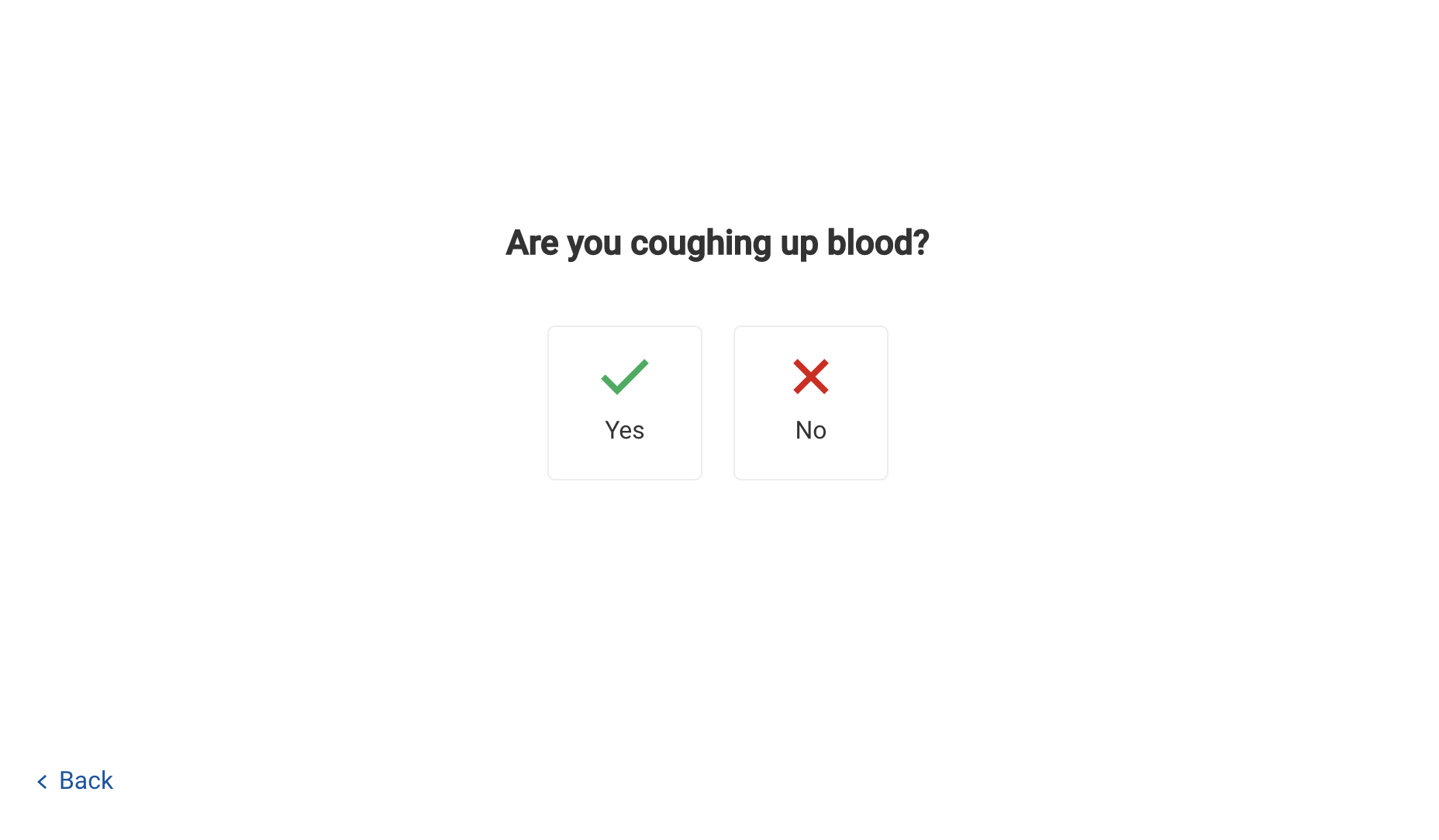
*

*3j. Screenshots of all questions provided in the tool- Coughing up blood*


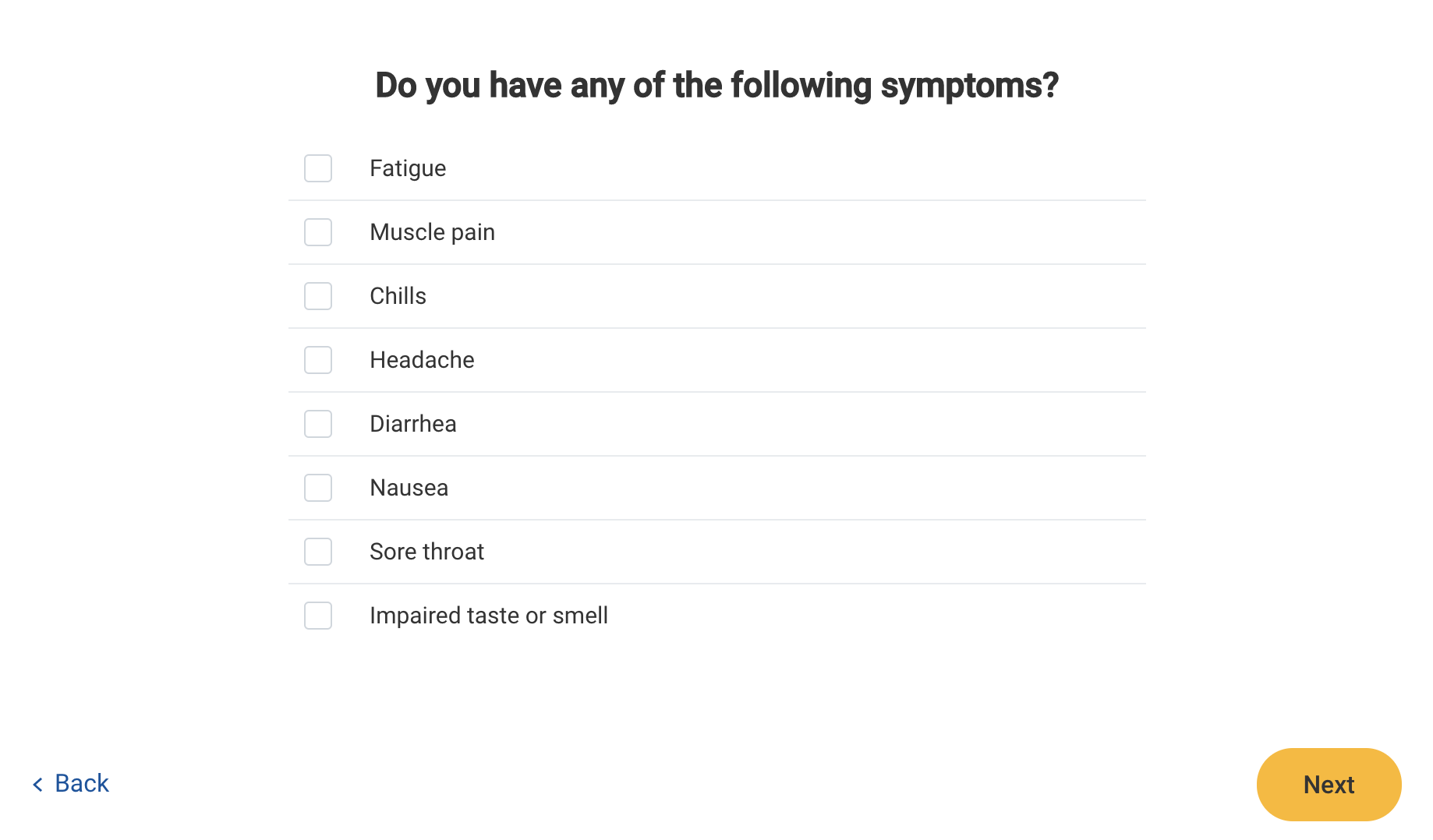


*3k. Screenshots of all questions provided in the tool- additional symptoms*

*
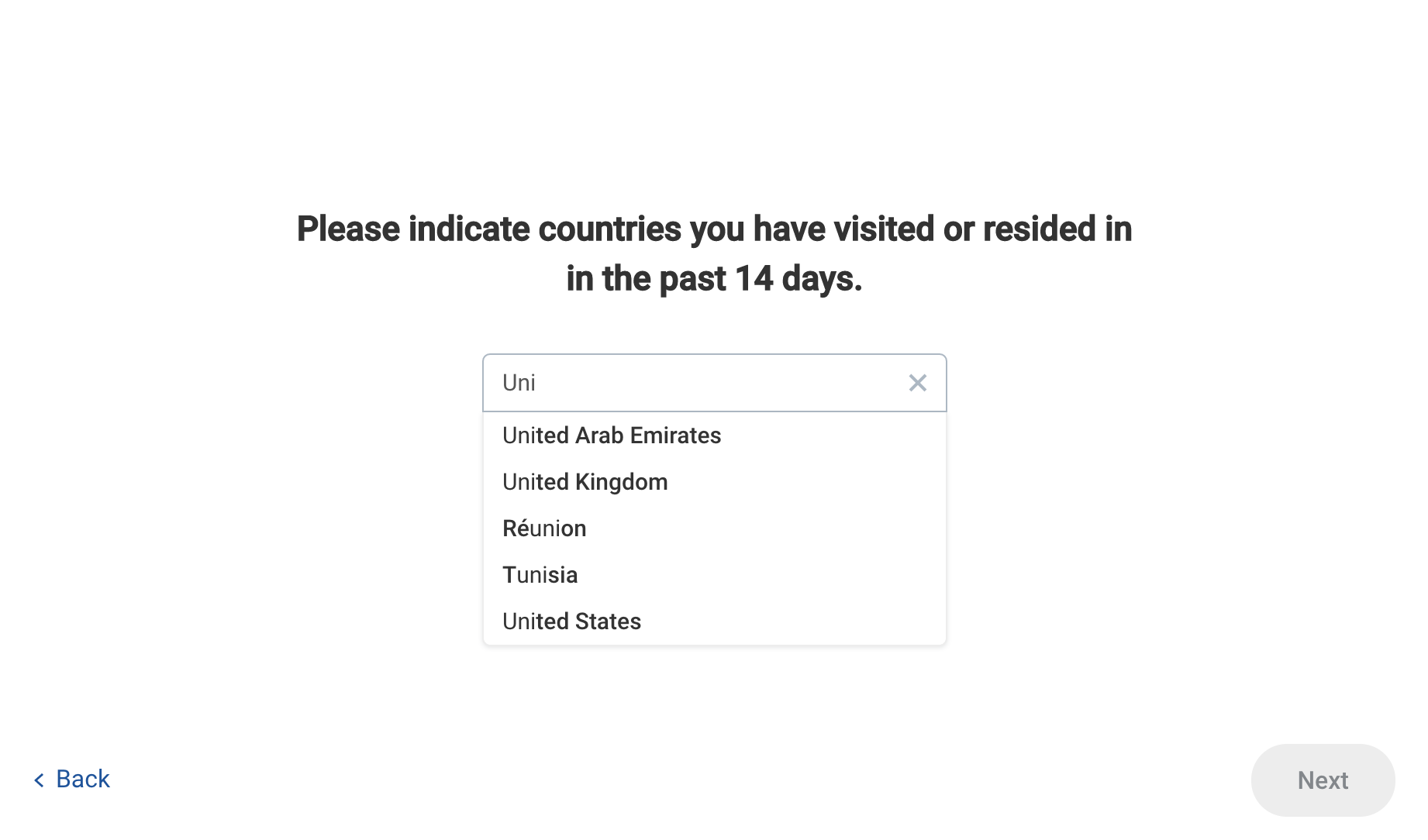
*

*3l. Screenshots of all questions provided in the tool- countries visited within last 14 days*

*
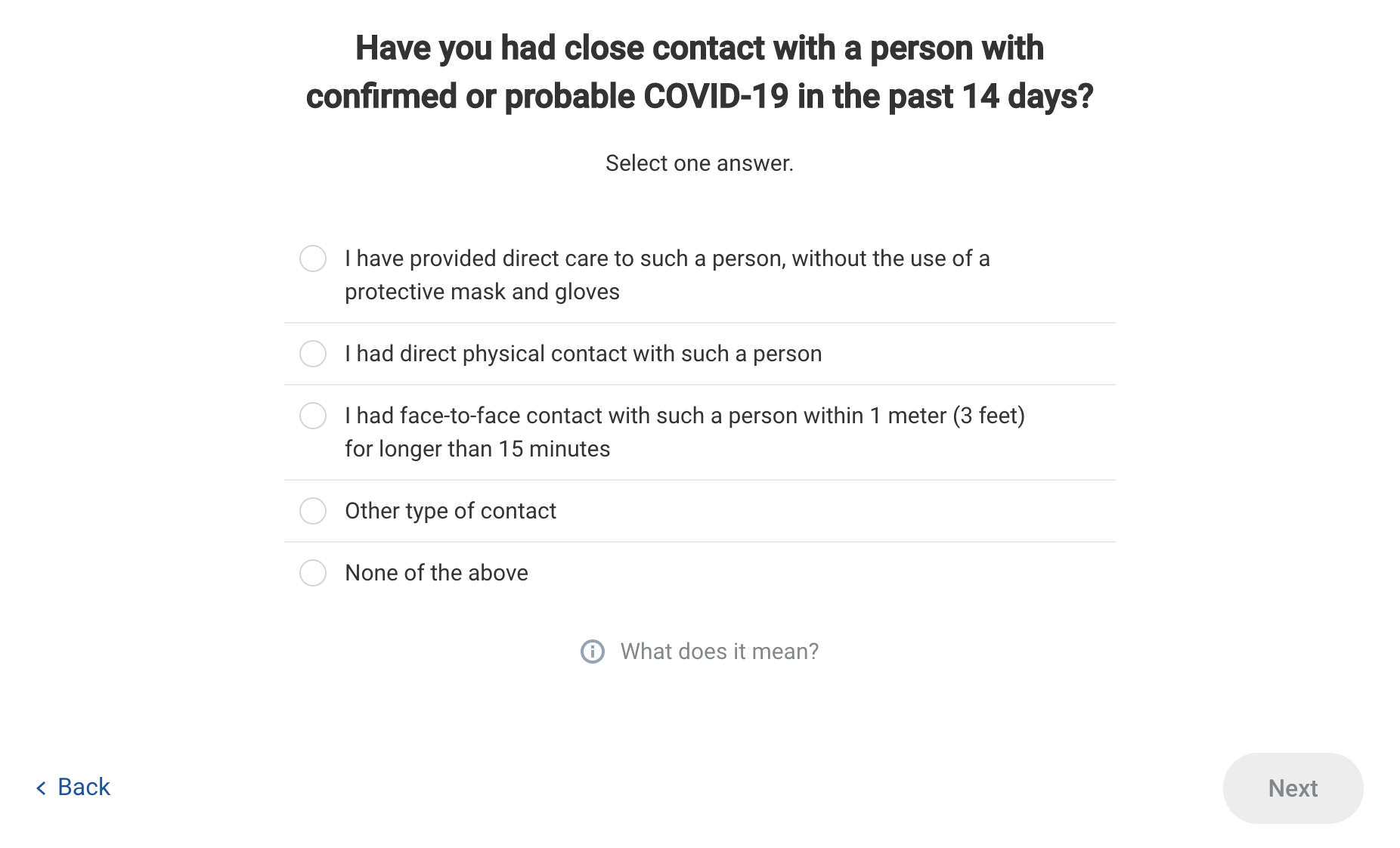
*

*3m. Screenshots of all questions provided in the tool- Assessing exposure to a confirmed COVID-19 case*


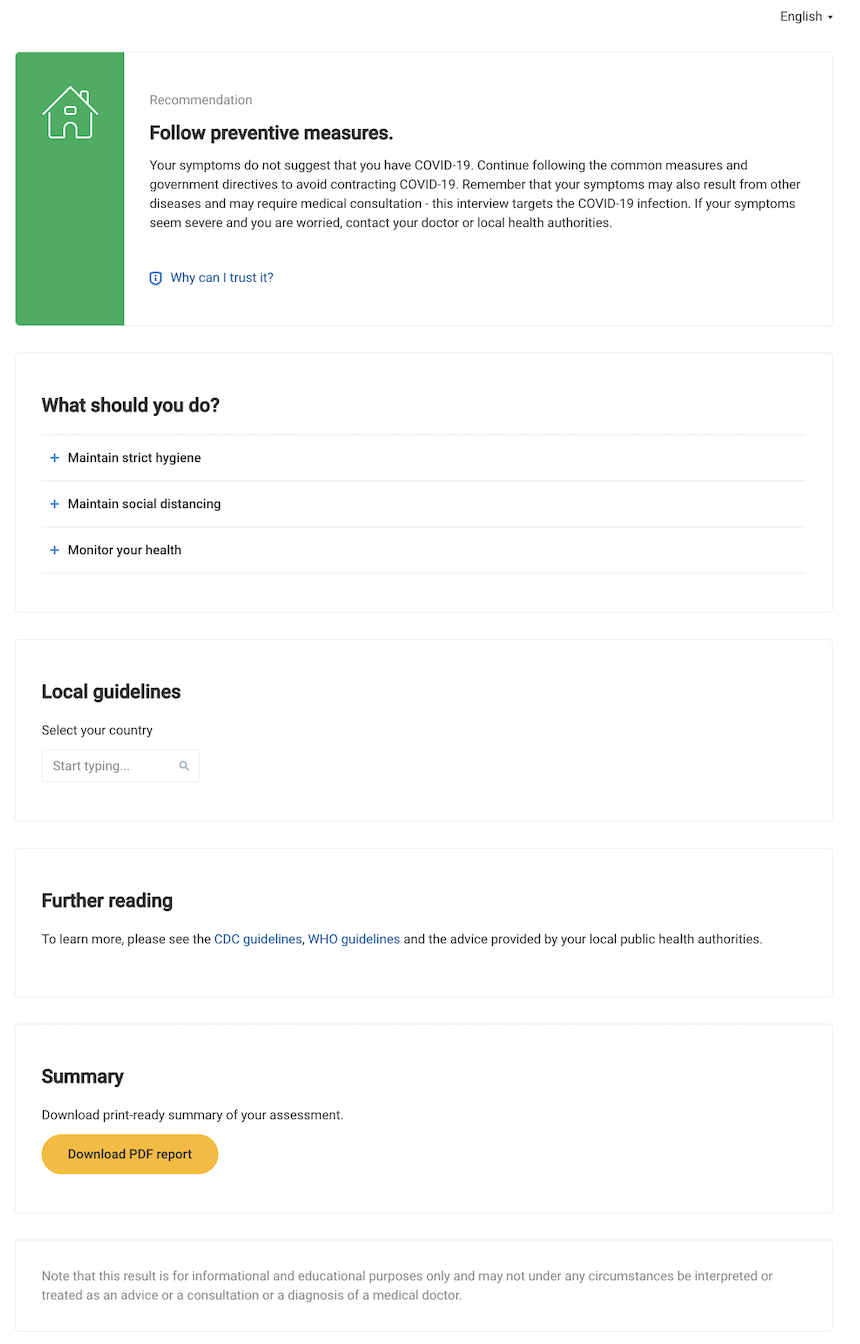


*3n Screenshots of all questions provided in the tool- Final screen for “Follow preventive measures” recommendation.*

*
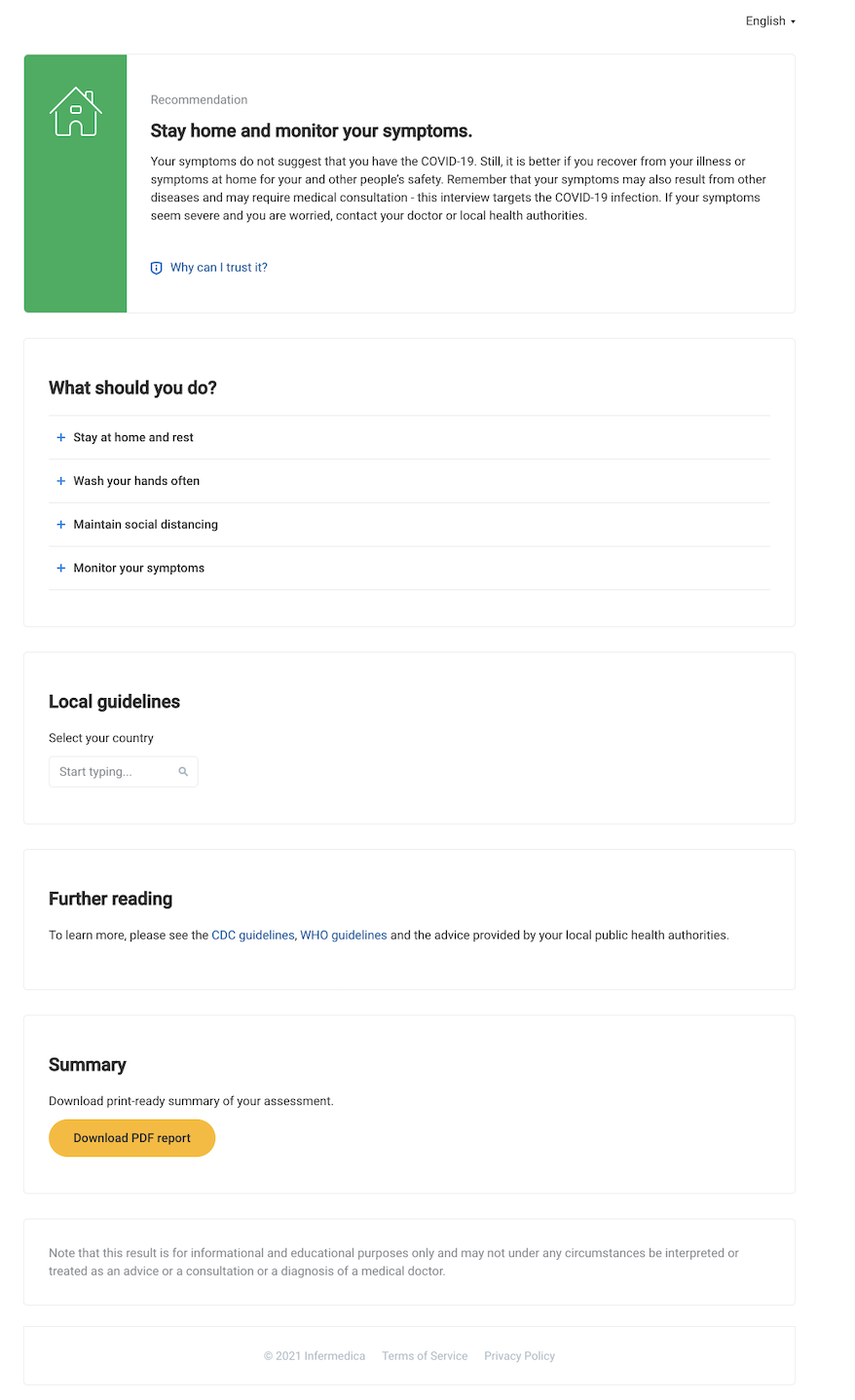
*

*3o Screenshots of all questions provided in the tool- Final screen for “Stay home and monitor your symptoms” recommendation.*

*
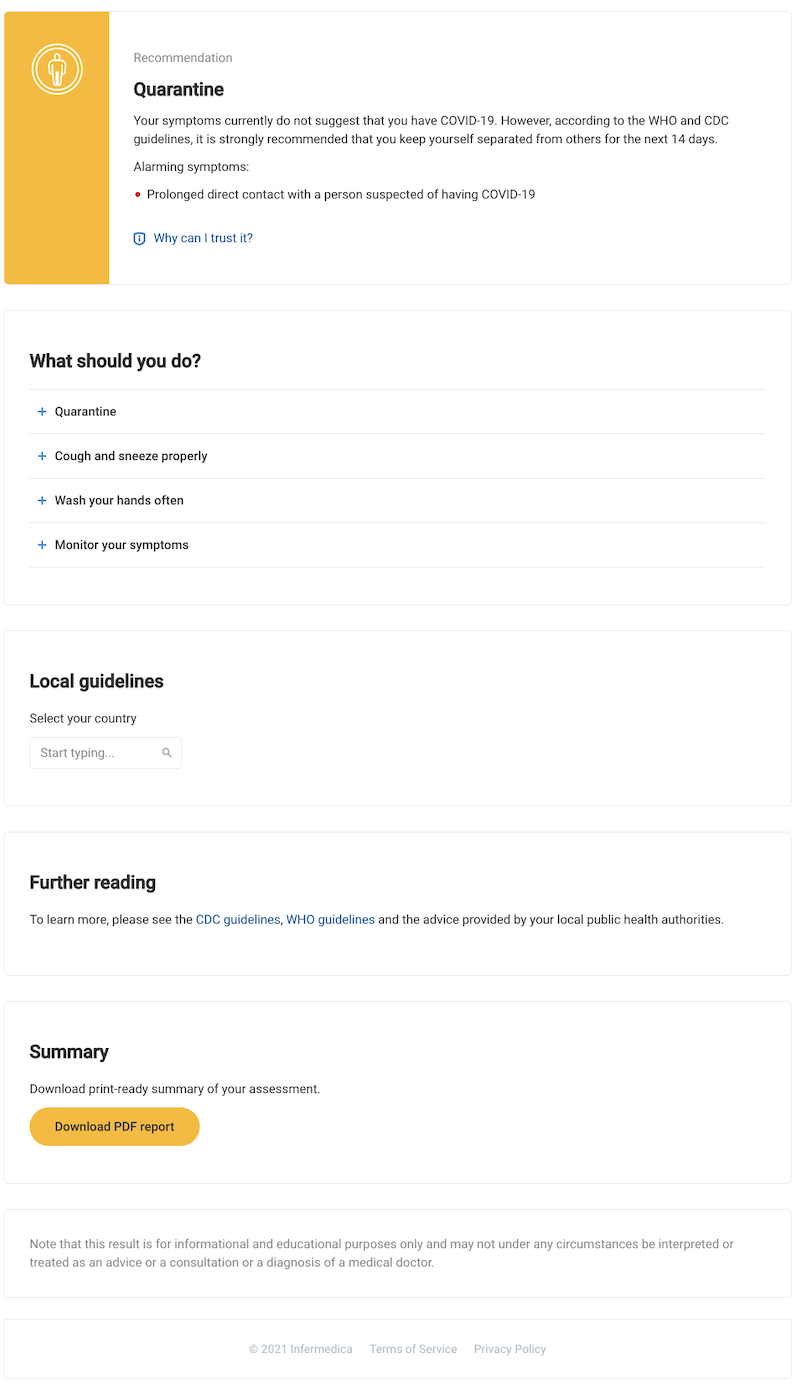
*

*3p Screenshots of all questions provided in the tool- Final screen for “Quarantine” recommendation.*

*
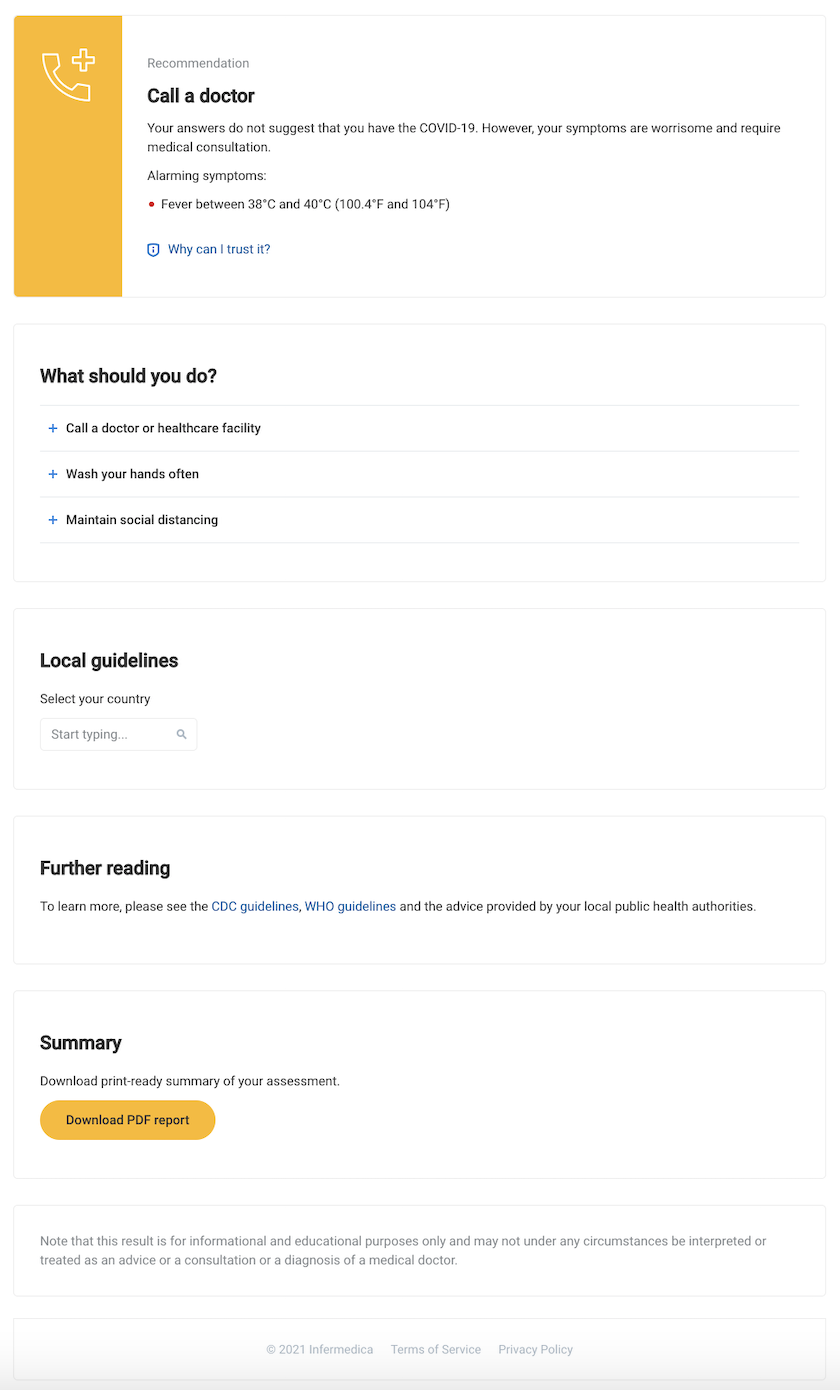
*

*3r Screenshots of all questions provided in the tool- Final screen for “Call a doctor” recommendation.*

*
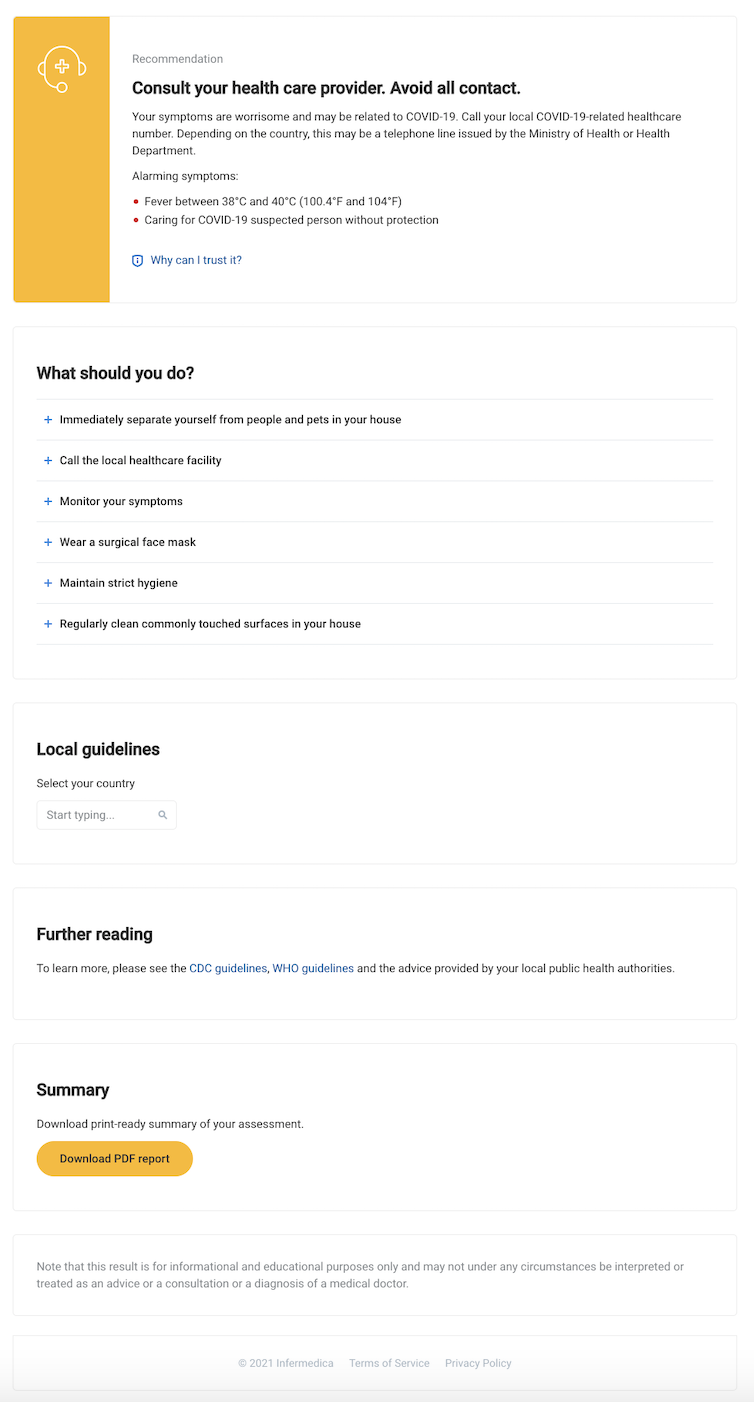
*

*3s. Screenshots of all questions provided in the tool- Final screen for “Consult your healthcare provider. Avoid all contact. recommendation.*

*
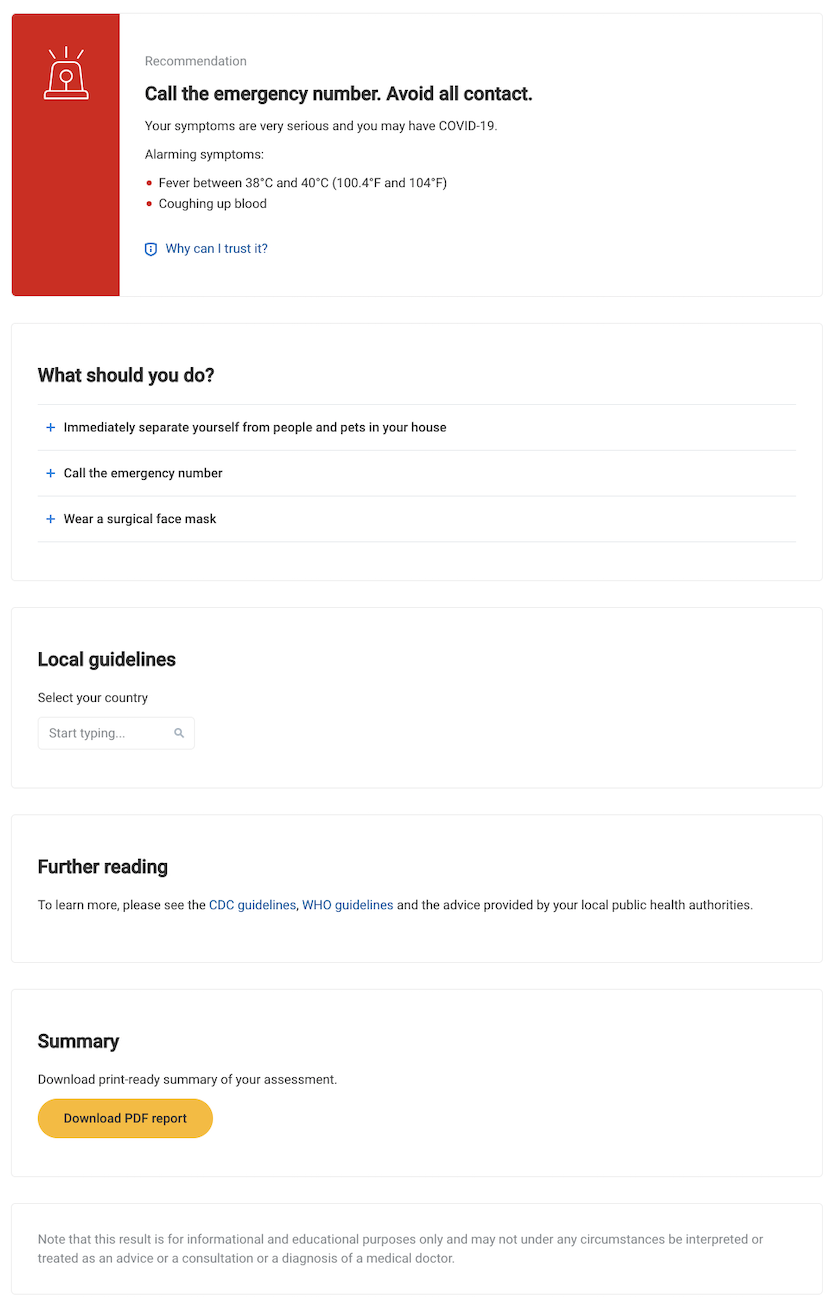
*

*3t. Screenshots of all questions provided in the tool- Final screen for “Call emergency number Avoid all contact. recommendation.*

*3. Risk factors screen with an explanation displayed.*
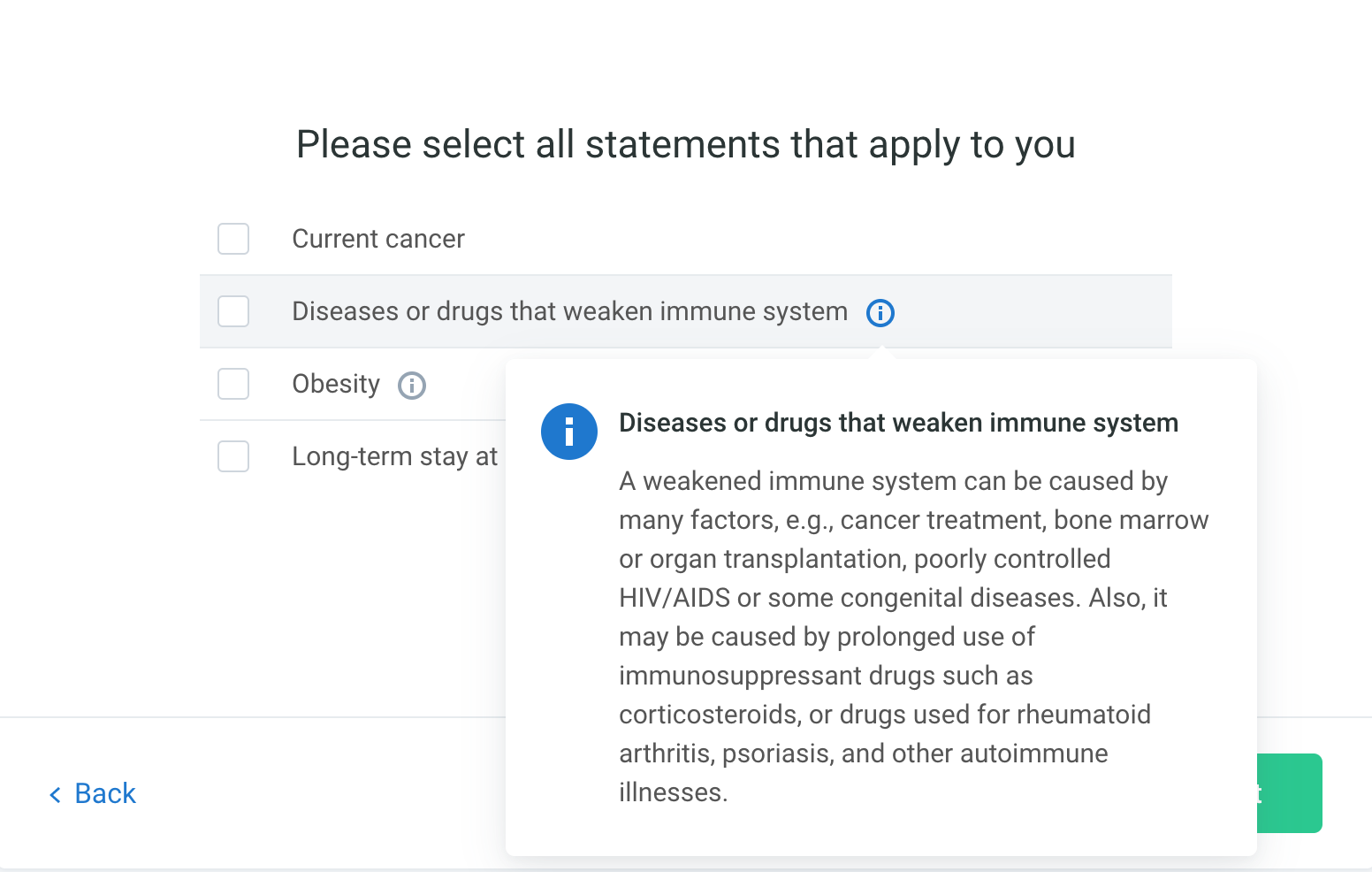


| Current cancer |  |
| --- | --- |
| Diseases or drugs that weaken immune system | A weakened immune system can be caused by many factors, e.g., cancer treatment, bone marrow or organ transplantation, poorly controlled HIV/AIDS or some congenital diseases. Also, it may be caused by prolonged use of immunosuppressant drugs such as corticosteroids, or drugs used for rheumatoid arthritis, psoriasis, and other autoimmune illnesses. |
| Obesity | A person is considered obese when his or her body mass index (BMI) exceeds 30. |
| Long-term stay at a care facility or nursing home |  |
| Diabetes |  |
| Cardiovascular disease | It’s a class of diseases affecting the heart and blood vessels. This condition: includes, e.g., heart failure, coronary artery disease, severe hypertension, or other heart diseases. Also, select this if you ever had a heart attack or stroke. |
| History of chronic lung disease | Chronic lung diseases are disorders that affect the lungs and other parts of the respiratory system. Disorders include Chronic Obstructive Pulmonary Disease, moderate and severe Asthma, and other lung conditions. |
| History of chronic liver disease | A long-term liver disease due to alcoholism, infections, chronic inflammation, or congenital diseases, leading to loss of function, cirrhosis and other complications. |
| History of chronic kidney disease | Chronic kidney disease is a gradual loss of kidney function to filter blood. It develops slowly over time, causing a waste build-up in your body. |

*4. Risk factors with the corresponding explanations.*

| Fever | Fever is an elevated body temperature above 37.5°C or 99.5°F. Select this symptom, even if you haven’t measured your temperature, but you feel hot, feverish, or seem flushed. |
| --- | --- |
| Cough |  |
| Shortness of breath | Shortness of breath: When you have trouble breathing and you cannot get enough air into your lungs. Often accompanied by chest tightness, breathlessness or a feeling of suffocation. |

*5. Major symptoms with explanations.*

| Follow preventive measures | Your answers do not suggest that you have COVID-19. Continue following the common measures and government directives to avoid contracting COVID-19. Remember that your symptoms may also result from other diseases and may require medical consultation - this interview targets the COVID-19 infection. If your symptoms seem severe and you are worried, contact your doctor or local health authorities. |
| --- | --- |
| Stay home and monitor your symptoms | Your symptoms do not suggest that you have the COVID-19 infection. Still, it is better if you recover from your illness or symptoms at home for your and other people’s safety. Remember that your symptoms may also result from other diseases and may require medical consultation - this interview targets the COVID-19 infection. If your symptoms seem severe and you are worried, contact your doctor or local health authorities. |
| Call a doctor. | Your answers do not suggest that you have the COVID-19. However, your symptoms are worrisome and require medical consultation. |
| Quarantine | Your symptoms currently do not suggest that you have COVID-19. However, according to the WHO and CDC guidelines, it is strongly recommended that you keep yourself separated from others for the next 14 days. If your symptoms seem severe and you are worried, contact your doctor or local health authorities. |
| Consult your healthcare provider. Avoid all contact. | Your symptoms are worrisome and may be related to COVID-19. Call your local COVID-19-related healthcare number. Depending on the country, this may be a telephone line issued by the Ministry of Health or Health Department. |
| Call the emergency number. Avoid all contact. | Your symptoms are very serious and you may have COVID-19. |

*5. Outcomes with recommendations*

| Immediately separate yourself from people and pets in your house | If possible, isolate yourself in a separate room, away from other members of your household. If this is not possible, keep a distance of at least 2 meters (6 feet) from others and wear a protective surgical mask. Avoid all interaction with household pets. For detailed instructions on how to set up an isolation space, visit [CDC guidelines](https://www.cdc.gov/coronavirus/2019-ncov/index.html). |
| --- | --- |
| Monitor your symptoms | Please monitor your health several times a day. If it is possible, keep a thermometer in your isolation place and monitor your temperature every couple of hours. Repeat this checkup if you notice any new symptoms.  When should you call 112 or your local emergency number?  You have trouble breathing.  Your fever climbs over 40°C, 104°F.  Your health is getting worse.  Tell health personnel that you are being evaluated for COVID-19. Put on a surgical face mask prior to their arrival. |
| Wear a surgical face mask | Put on a surgical face mask whenever somebody is about to enter the same room or vehicle as you. Put on a surgical face mask before entering a medical facility. People interacting with you should wear face masks as well, particularly if you have difficulty breathing. |
| Maintain strict hygiene | It is strongly recommended to take simple precautions that can reduce your chances of becoming infected in the future or of spreading the virus:  Regularly and thoroughly clean your hands with an alcohol-based hand sanitizer or wash them with soap and water for at least 20 seconds.  Avoid touching your eyes, nose and mouth.  Cover your mouth and nose with your bent elbow or tissue when you cough or sneeze.  Disinfect your hands after sneezing.  Do not share your cups, plates, cutlery and other household utensils with family members. |
| Regularly clean commonly touched surfaces in your house | Surfaces and items like doorknobs, bathroom fixtures, toilets, phones, keyboards, tablets, and bedside tables easily get contaminated with the coronavirus. Clean them regularly with household cleaners, to avoid transmitting the disease to other household members. Pay extra attention to the toilet area and other places that may have residue bodily fluids on them. Cleaning guidelines can be found here. |
| Quarantine | Choose a well-ventilated single room, or if a single room is not possible, please maintain a distance of at least 2 meters (or 6 feet) from other household members and minimize the usage of shared spaces like a kitchen or bathroom. Separation should last for 14 days, counting from the last day of exposition to the virus (in some countries the duration of quarantine may differ).  For detailed instructions on how to set up a quarantine space, visit CDC guidelines. |
| Cough and sneeze properly | Cover your mouth and nose with a tissue when you are about to cough or sneeze. Throw the used tissue out into a bin lined with a trash bag. Disinfect your hands afterward with an alcohol-based rub containing a minimum of 60% of alcohol, or wash your hands with soap and water for a minimum of 20 seconds. |
| Wash your hands often | Your hands are a carrier for the coronavirus. Every time you touch your face area, cough into your palms, or go to the toilet, your hands become contaminated and spread the virus to everything you come into contact with. It is crucial, for the safety of your household members, that you clean your hands regularly with soap and water or an alcohol-based rub. |
| Keep social distance | Maintain at least 2 meters (6 feet) distance between yourself and other people, especially when they are coughing or sneezing.  Stay at home, especially if you are an older person or have diabetes, heart, or lung disease. |

*6. Post-outcome recommendations*

| Date of the consultation [DD/MM/2020] | No of patients examined/day |
| --- | --- |
| 07/04/ | 76 |
| 08/04 | 81 |
| 09/04 | 68 |
| 10/04 | 74 |
| 13/04 | 26 |
| 15/04 | 29 |
| 30/04 | 19 |
| 06/05 | 25 |
| 12/05 | 21 |
| 27/05 | 20 |
| 04/06 | 12 |
| 10/06 | 16 |
| 17/06 | 11 |
| 24/06 | 11 |
| 03/07 | 22 |
| 08/07 | 22 |
| 15/07 | 13 |
| 23/07 | 13 |
| 29/07 | 24 |
| 06/08 | 18 |

*7. A number of consultations in the Admission Room per day on a given date*

| Centrum Systemów Informacyjnych Ochrony Zdrowia- Polish Ministry of Health |
| --- |
| Ministry of Health of Ukraine |
| Global Excel |
| PZU Zdrowie |
| Dovera |
| Batist Medical |
| International Medical Center Hospital |
| Gdzie Po Lek |
| Dr Sintomas |

*8. List of companies that implemented the Covid-19 Risk Assessment tool (ones that approved sharing publicly information about their participation)*
